# Helical nanostructures for organic electronics: the role of topological sulfur in *ad hoc* synthesized dithia[7]helicenes studied in the solid state and on a gold surface†

**DOI:** 10.1039/d0na00045k

**Published:** 2020-03-16

**Authors:** Bianca C. Baciu, Tamara de Ara, Carlos Sabater, Carlos Untiedt, Albert Guijarro

**Affiliations:** Departamento de Química Orgánica, Instituto Universitario de Síntesis Orgánica, Unidad asociada al CSIC, Universidad de Alicante Campus de San Vicente del Raspeig E-03080 Alicante Spain aguijarro@ua.es; Departamento de Física Aplicada, Unidad asociada al CSIC, Universidad de Alicante Campus de San Vicente del Raspeig E-03080 Alicante Spain untiedt@ua.es

## Abstract

As the first of a series of molecular solenoids, two classes of dithia[7]helicenes (coil-shaped molecules with sulfur atoms integrated within a helical conjugated system) have been devised and synthesized to be used in molecular electronics. We used a modular assembly of fragments using Pd catalyzed coupling reactions and a final photocyclization step for the syntheses; this strategy gave us straightforward access to helicenes bearing thiophene end rings with either *exo* or *endo* topologies. Unequivocal structural characterization was carried out by X-ray crystallography. In the solid state, their crystal architectures show little similarities; both can be considered an ensemble of heterochiral dimers (*P*/*M*) that are themselves very different in nature in light of their main pairing interactions. On a gold surface, the effect of the sulfur atom is to strengthen their binding to the electrodes, as manifested by scanning tunneling microscopy (STM) performed at room temperature. Different coating patterns were observed for each class of molecule, although the most prominent finding is that we could see resolved STM images of a single molecule, with a full display of its inherent chirality under room temperature conditions.

## Abstract 2

As the first of a series of molecular solenoids, two classes of coil-shaped molecules (dithia[7]helicenes) have been designed and synthesized to be used in molecular electronics.

## Graphical abstract

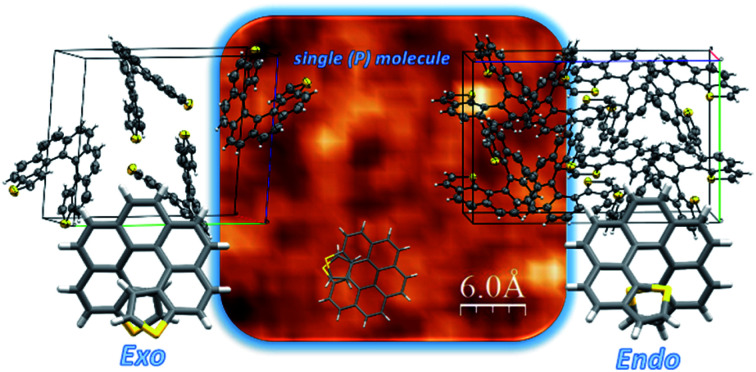

## Introduction

**Fig. 1:**
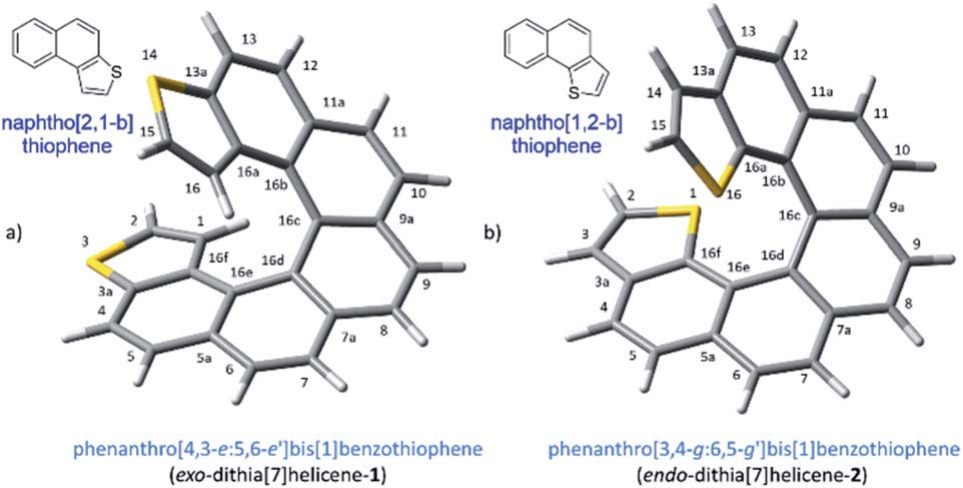
Chemical structure, name and numbering of the dithiahelicenes considered in this work: (a) *exo*-dithia[7]helicene-1, and (b) *endo*-dithia[7]helicene-2. Details of the thiophene type of ring fusion endowing an *endo* or an *exo* arrangement of the sulfur atoms with respect to the helix (top left insets). Alternative names using specific nomenclature for heterohelicenes are 3,14-dithia[7]helicene (1) and 1,16-dithia[7]helicene (2).

The demand for nanosized electronic devices intended to be used in molecular electronics calls for important improvements in the molecular design, necessary to overcome the numerous challenges met at this scale in a realistic scenario. Adequate performances under normal operating conditions, such as room temperature, require a strengthened binding interaction with metallic electrodes while preserving the conduction properties of the device. Charge-transport properties of a single molecule device are influenced by several intercorrelated elements, the electrode material, the contact interface and the molecule itself, which is the most easily tunable part of the ensemble by chemical means.^1^ Helicenes,^2^ are likely the first choice if the intention is to build a molecular solenoid that could offer experimental evidence of the chiral-induced spin selectivity effect (CISS)^3^ through electronic transport measurements. These molecules have a robust helical architecture that is expected to display excellent conduction properties despite its non-planarity as a result of the still highly conjugated electronic π system.^4^ The topologies of adsorption on a metallic surface of carbohelicenes have been well described. The three rings at the ends of a helicene are rather flat and accessible to a metallic surface, resting on them and spiraling away from the surface beneath.^5^ Several carbohelicenes have been mapped using scanning tunneling microscopy (STM)^6^ revealing this type of weak binding between the molecule and the surface.^7^ However excessive thermal molecular mobility prevents visualization of any ordered structure above 60 K.^5^ In our efforts to overcome any shortcoming due to weak binding interactions and advance towards better nanoelectronic devices, we have considered a helicene type of molecule with fused thiophene rings at the ends. Integrating sulfur atoms within the terminal ring (rather than as a pending functional group) maintains the overall π conjugation of the molecule, while strengthened interactions with the metal electrode are expected to be developed, particularly at the terminal rings where thiophene motifs are located. This approach led us to two types of molecular layouts, what we call *endo* and *exo*-dithia[7]helicenes for simplicity, based respectively on the naphtho[1,2-*b*]thiophene and naphtho[2,1-*b*]thiophene type of ring fusion (Fig. 1). The number of sulfur atoms was deliberately restricted to two. Polarization exerted by the presence of additional electronegative sulfur atoms in the helical circuit other than in the necessary terminal junctions might have a deleterious effect on the performance of the device affecting negatively the electrical conductance of the ensemble, also allowing an unwanted scenario of multiple binding configurations on gold. In this paper, we undertake a multidisciplinary task studying the topologies and nature of the interactions of these di-terminated heterohelicenes both in the solid state as well as on metallic gold as a preamble to studying their conduction properties. It is organized as follows: in Section (1) we propose and carry out an efficient synthesis of both molecules, in (2) we characterize their crystal structures and analyze their packing interactions, and in Section (3) we perform an STM study on deposition *via* drop casting and spin-coating on a gold surface at room temperature.

## Results and discussion

### Synthesis of *endo* and *exo*-dithia[7]helicenes 1 and 2

**Scheme 1:**
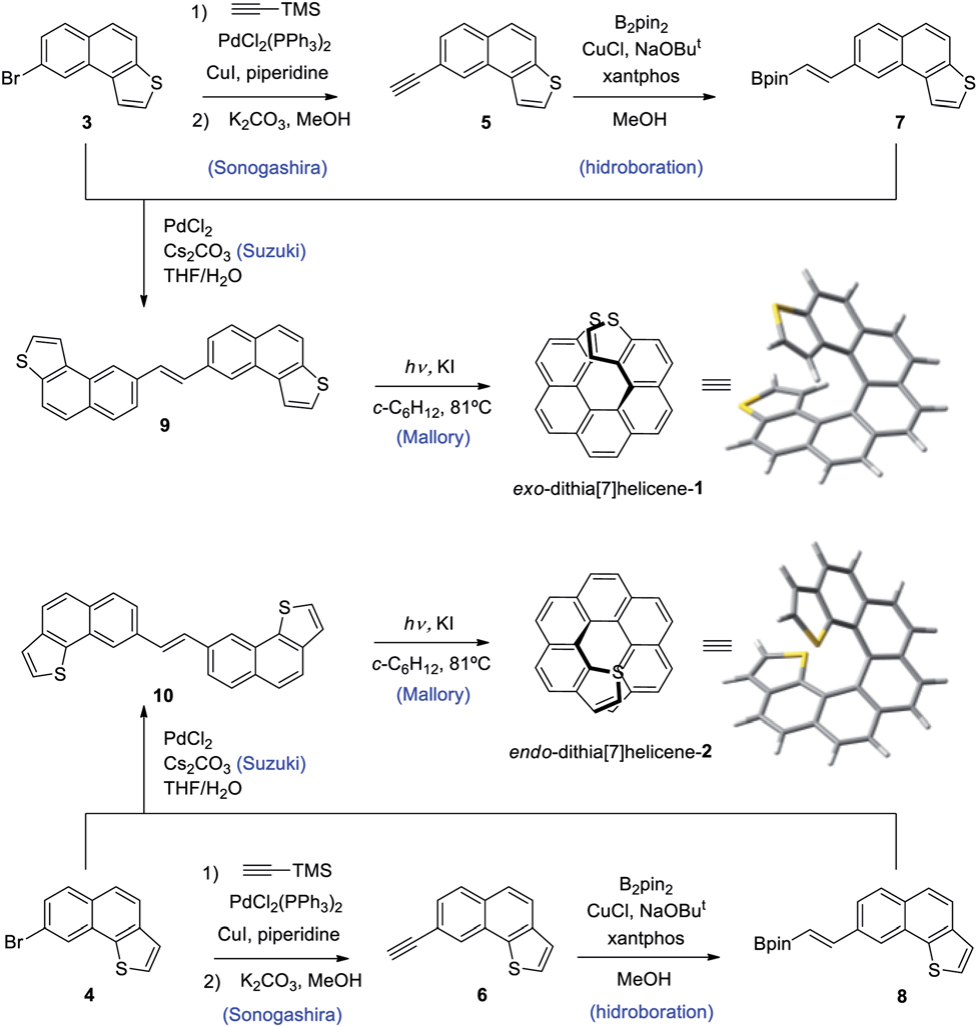
Synthesis of *exo* and *endo*-dithia[7]helicenes 1 and 2.

Our synthetic scheme adapted state of the art methods of Pd catalyzed coupling reactions with the pioneering work on the photochemical synthesis of thiahelicenes by Wynberg and coworkers,^8^ which inspired the key step of this synthetic strategy. It was already evident back at that time the great difficulty encountered in obtaining naphtho[1,2-*b*]thiophene derivatives (with *endo* S atom) in comparison to naphtho[2,1-*b*]thiophene ones (with *exo* S atom) by means of a photocyclization route.^9^ Indeed, to date, a large number of thiahelicenes including di,^10^ tri, tetra, and hexathia[7]helicenes have been synthesized in this manner, always bearing S atoms in *exo* position.^11^ To the best of our knowledge, there is however only one example of an *endo* thiahelicene, namely 1-thia[7]helicene, synthesized only recently from 1-helicenethiol by construction of a new fused thiophene ring attached to the sulfur predetermined position.^12^ With this background in mind, we carried out first the synthesis of the fragments 3 and 4, bearing the desired naphtho[2,1-*b*]thiophene and naphtho[1,2-*b*]thiophene ring fusion, as precursors of *exo*- and *endo*-dithia[7]helicene-1 and 2, respectively (Scheme 1, see the ESI† for an efficient synthesis of 3 and 4). Ethynylation of 3 and 4 was achieved by means of the Sonogashira reaction using (trimethylsilyl)acetylene followed by desilylation in a basic medium to afford 5 and 6, respectively. Hydroboration of 5 and 6 provided the vinylboronic derivatives needed for a subsequent Suzuki coupling with 3 and 4. This was done by means of bis(pinacolato)diboron through a copper catalyzed reaction activated by bidentate diphosphine ligand xantphos,^13^ which afforded the (*E*)-vinylboronate 7 and 8 in good yields. Suzuki coupling of 7 and 8 with the original fragments 3 and 4 respectively gave rise to the desired stilbenic precursors 9 and 10, ready for the photocyclization step of the Mallory reaction. One of the key factors to success in our synthesis was the implementation of high temperature conditions during irradiation, which was carried out in refluxing cyclohexane, thus increasing the limited solubility of the stilbenic precursors. This, along with a simple photocyclization protocol using KI as a catalyst and air as the ultimate oxidant for the aromatization step,^14^ afforded the desired dithiahelicenes. Another key factor was a modular type of assembly, based on the preformed building blocks 3 and 4. This allows us to use a single synthetic scheme for the two target molecules, whose topologies have been irrefutably verified by X-ray diffraction. Synthetic details of the procedures of Scheme 1 are fully described in the ESI.†

### Crystal structures of *endo* and *exo*-dithia[7]helicenes-1 and 2

**Fig. 2:**
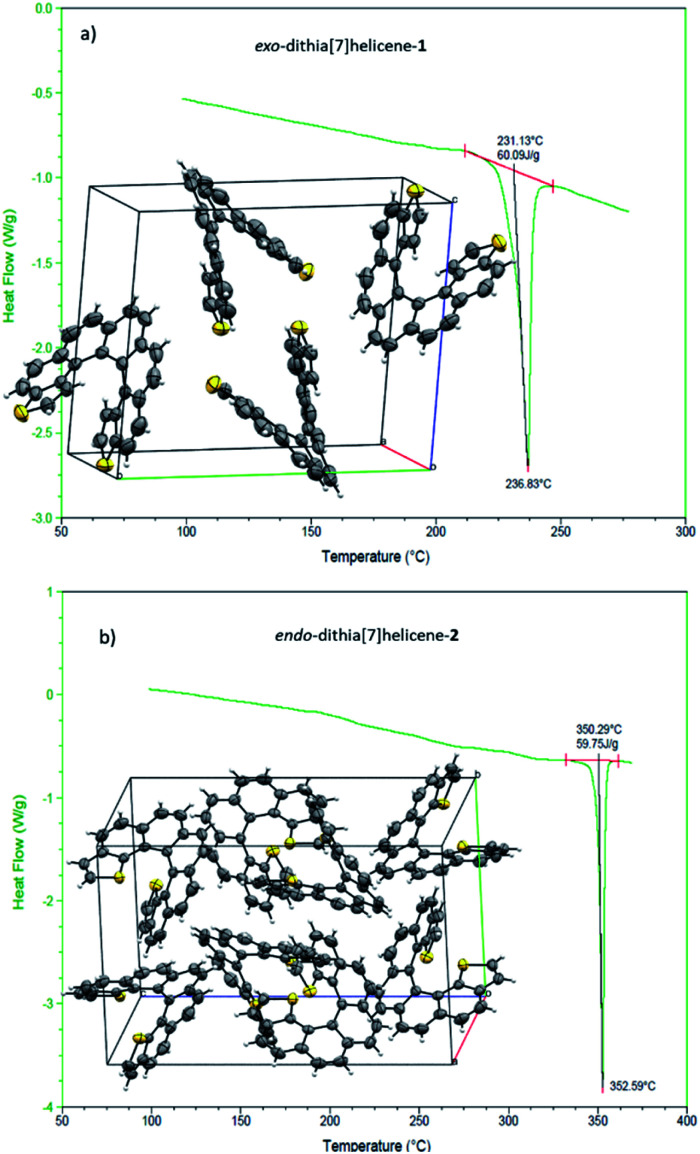
Crystal structure and differential scanning calorimetry results (DSC) of: (a) on top, *exo*-dithia[7]helicene-1: Monoclinic unit cell, space group *P*2_1_/*c*, *a* = 8.5983(7), *b* = 15.8995(12), *c* = 13.9577(11) (Å); *α* = 90, *β* = 102.844(2), *γ* = 90°; *Z* = 4. DSC shows a sharp endothermic event corresponding to melting. A mp = 231.1 °C is given by the extrapolated onset temperature of the melting peak. (b) At the bottom, the crystal structure of *endo*-dithia[7]helicene-2: orthorhombic unit cell, space group *Pbca*, *a* = 13.5378(10), *b* = 13.3994(9), *c* = 20.4288(15) (Å); *α* = *β* = *γ* = 90°; *Z* = 8. DSC shows another sharp endothermic event corresponding to melting with some (negligible) decomposition. A mp = 350.3 °C is given by the extrapolated onset temperature of the melting peak. For the crystal cells, ORTEP diagrams are shown with thermal ellipsoids drawn at 50% probability and data are acquired at 298 K.

We then turned to the study of the structure of 1 and 2 in the solid state in order to gain some understanding on the preferred topologies of aggregation that could be displayed by these helicenes. Monocrystals of adequate size and quality for X-ray diffraction analysis were obtained from saturated ethyl acetate solutions by slow evaporation at room temperature. Under these conditions, both isomers crystallize as racemic crystals; 1 in the monoclinic *P*2_1_/*c* and 2 in the orthorhombic *Pbca* space groups, with *Z* = 4 and 8 molecules in the cell respectively (Fig. 2). There are two particularities in these crystals that merit structural analysis in detail. First, their strikingly similar density (less than a 0.5% of difference) despite displaying very different packing designs. Secondly, a dramatic difference in melting points (of about 120 °C) in spite of their similar packing efficiency as evidenced by the densities (DSC analyses in Fig. 2). A closer look at the crystal packing forces should give us further information on these basic properties; for comparison, racemic [7]helicene has been included in the study.^15^ There is only one type of symmetrically independent molecule in both crystals, in *exo*-1 and also in *endo*-2; however since they belong to a centrosymmetric point group (*i.e.* they are racemic crystals) they come in pairs of enantiomers, just as *rac*-[7]helicene, which belongs to the *P*2_1_/*c* space group as well. Other than that, there is no further resemblance in the crystal packing architecture for these three closely related racemates. Association in the form of heterochiral (*P*/*M*) dimers is an energetically favored topological motif in [7]helicene, which has been observed adsorbed on copper at low temperature.^16^ As a starting point, we have looked for the closest interacting pair of molecules in the crystal, which can be considered a basic building block of the network.

**Fig. 3:**
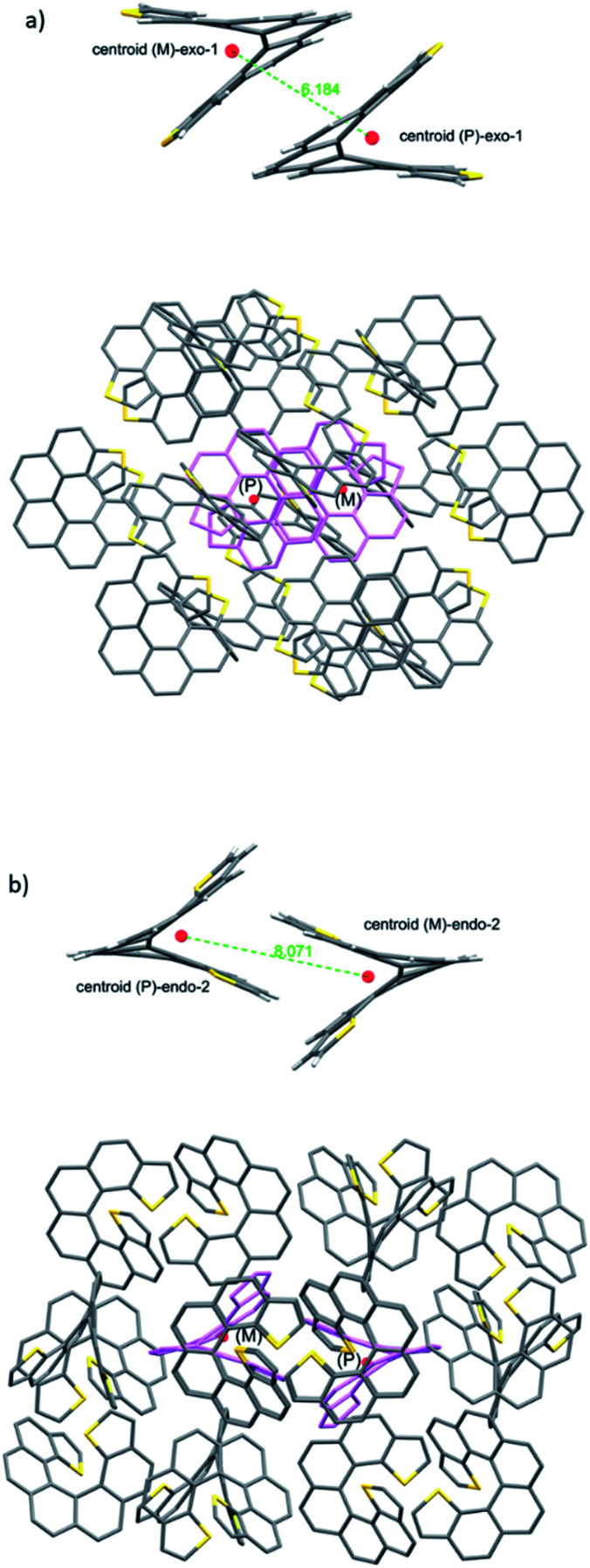
Basic building units of the crystals formed by heterochiral (*P*/*M*) pairs of molecules: (a) on top, the closest interacting pair of centroids of the *exo*-dithia[7]helicene-1 crystal showing a face-to-face type of interaction between a *P*/*M* pair of molecules. At the bottom, a shell of 16 first neighbors surrounding the heterochiral pair shown above (tinted in pink). (b) The same for *endo*-dithia[7]helicene-2 showing a bite type of interaction, and a shell of 18 neighbours surrounding it (also tinted in pink).

*Exo*-1 has a heterochiral pair with only 6.184 Å separating the centroids of the *P* and *M* dithia[7]helicene molecules (Fig. 3a, top). This pair interacts in a face-to-face fashion using two central rings (containing the carbons from 5a to 9a and 16c–16e, Fig. 1), with a mean plane separation distance of 3.943 Å for this π–π interaction (see Fig. 3a, bottom and S1 in the ESI†). This racemic pair is surrounded by a shell of 16 molecules among which 8 edge-to-face (mainly S-π type) and 4 bite type interactions can be distinguished. The remaining 4 molecules come in two pairs of units identical to the central pair that collectively forms an array of enantiomeric pairs in the *c* direction, the shortest cell axis (see Fig. 3a, bottom and experimental cif). The melting point of *exo*-1 is mp = 231.1 °C (Fig. 2). In spite of the closeness between the two enantiomeric centers in the dimer, the cell-calculated crystal density of *exo*-1, *d* = 1.394 g cm^−3^, is very similar to that of *endo*-2, that displays however a completely different set of interactions.

The building unit of crystal *endo*-2 is another heterochiral pair in which centroids are separated by 8.071 Å, interacting through a bite type of interaction (Fig. 3b, the top). This specific interaction is composed of one slight π–π overlap between the terminal thiophene rings of both molecules (mainly through carbon 3 in both molecules, Fig. 1), along with two clear CH–π interactions at 2.789 Å between the opposite, nearly perpendicular thiophene rings (CH of carbon 3 and the opposite thiophene ring, see also Fig. S2 in the ESI†). This racemic pair is surrounded by a shell of 18 molecules, the vast majority of which interact through an edge-to-face interaction of the CH–π type. In eight of them the central dimer plays the role of an acceptor, in the other eight the role of a donor, and for the last two molecules there is not a well-defined role (Fig. 5b, bottom and experimental cif). Contrarily to 1, there are no face-to-face interactions between molecules. The calculated crystal density of *endo*-2 is however *d* = 1.400 g cm^−3^, very close to that of 1 despite the differences in packing, while its melting point is much higher, mp = 350.3 °C (Fig. 2).

From our studies on the crystal architecture of PAHs and in particular on phenacenes which are electronically much related to helicenes, the major contribution to reticular energy is provided by the CH–pi interaction, which is additive and responsible for the typical herringbone arrangement displayed by these PAHs.^17^ This type of interaction seems to be an overwhelmingly dominant motif in *endo*-2, endowing it with a high melting point. On the other hand, the presence of an *exo* sulfur in 1 precludes this, at least to some extent, fostering the emergence of other types of apparently less robust interactions, such as S–π and other less defined ones. For comparison, in the case of *rac*-[7]helicene,^15^ the closest interaction (7.932 Å) between the heterochiral pair is non-specific in nature (neither clearly face-to-face nor CH–π), which along with the particularities of the surrounding shell makes it another type of packing manifestly different from 1 and 2.

### STM imaging of *exo* and endodithia[7]helicene-1 and 2

In order to study the anchoring of the molecule to the surface and its geometrical properties, such as chirality and size, Scanning Tunneling Microscopy is the best technique due to its subnanometric resolution even under ambient conditions. We want to highlight the importance of the identification of the chirality and size of the molecules for their implications in molecular electronics, specifically their role in a single-molecule spin-filter device.^18^ Topographic images were acquired applying a bias voltage in the range of [0.3–0.5] V to the tip (Pt_90_Ir_10_) in a constant current mode. The current amplification was of 10^9^ V/A. In order to gain resolution to distinguish the molecules, we have also used the Continuous Imaging Tunneling Spectroscopy (CITS) approach, which allows the acquisition of energy-resolved images.^19^

*Exo* and *endo*-dithia[7]helicene-1 and 2 were deposited on gold (111) surfaces from a benzene solution in different concentrations.^20^ Two methodologies were used for the deposition of the molecules: drop casting and spin coating. In the case of the drop casting deposition, the molecular solution over the gold was dried blowing argon gas and through vacuum (10^−3^ mbar). For the spin coating method, the drying process is not required, as the high-speed rotation removes the solvent. Overall, the results obtained show no significant difference between the two methods.

**Fig. 4:**
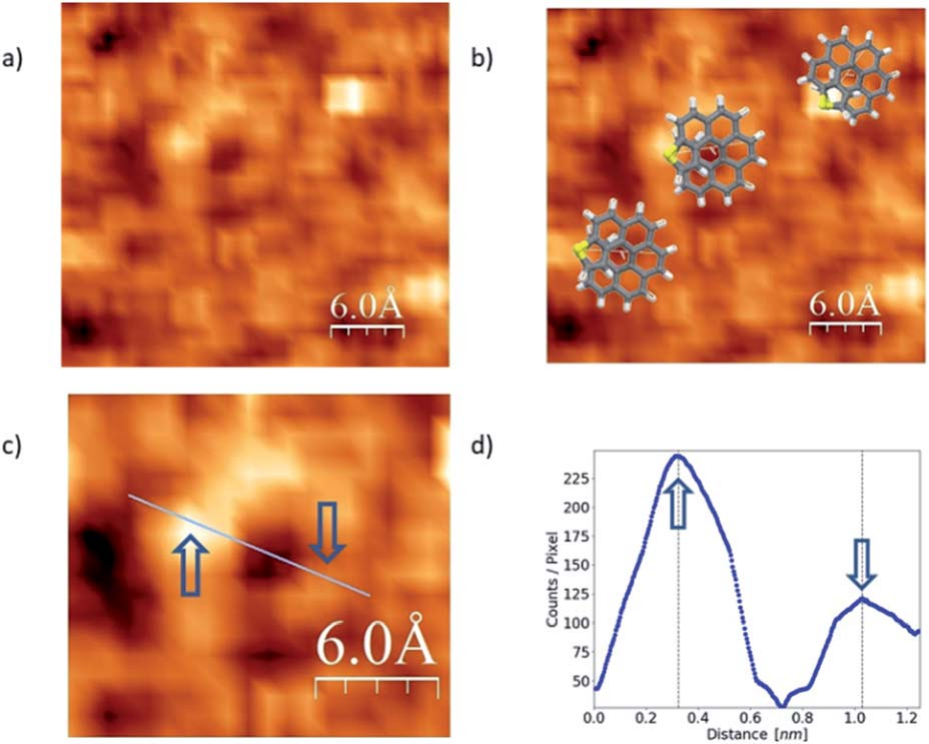
Panel (a) shows *exo*-dithia[7]helicene-1 molecules deposited *via* the spin coating method. Image acquired at *V*_bias_ = 0.6 V. Overlapped molecular models are shown in panel (b). Panel (c) shows a zoomed in image of a single molecule. Panel (d) shows the molecule profile, perpendicular to the surface as shown in (c).

In the literature we found reports on the identification of single helicene molecules only for UHV and low temperature conditions.^7,16^ Unexpectedly, for the case of our *exo*-dithia[7]helicene molecules we have acquired helicene single-molecule STM-images, under ambient conditions (Fig. 4). This observation of single molecules under ambient conditions could be due to the role of S atoms as anchoring sites of the molecule. However, it should be mentioned that for low concentrations we are able to distinguish the solvent molecules that may improve the visualization of the helicene molecules.^21^ Other authors^22^ have pointed out to the influence of solvents in the STM observation of single molecules at ambient conditions. Although this second reason may influence our results, we saw differences in between *endo* and *exo* molecules that can only be explained if the sulfur atoms play a role as anchoring sites for the molecule.

The STM image in Fig. 4 was acquired *via* CITS from a 0.2 mg ml^−1^ concentration and deposited using spin-coating. Panel (a) shows single molecules lying flat over the surface, whereas overlapped illustrations of the molecules are displayed in panel (b). A zoomed in image of a single and isolated molecule is shown in panel (c), and a topographic profile in panel (d). Blue arrows indicate where the profile was measured. From the images we can obtain the main topographic characteristics of the molecules. We have identified a diameter of about 0.70 ± 0.02 nm as expected from the molecular geometry (see Fig. S2f in the ESI†). On the other hand, from the image and profile characteristics we were able to identify the molecule chirality, which in this case is *P*.

**Fig. 5:**
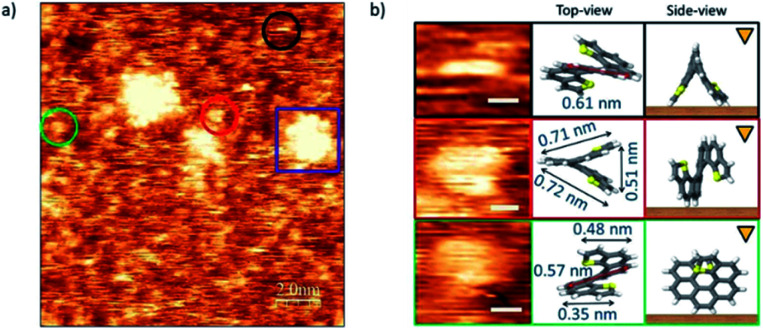
Topographic images of *endo*-dithia[7]helicene-2 molecules acquired at room temperature in a concentration of 0.2 mg ml^−1^ deposited by the spin coating method. (a) Topographic image. The bias parameters are *I*_*t*_ = 1 nA and *V*_bias_ = −0.4 V. (b) Zoomed in image of different positions the molecule adopts when deposited. Topographic image, top and side view representations are shown. The scale bar represents 0.3 nm.

The case of *endo*-dithia[7]helicene molecules deposited by spin coating in a concentration of 0.2 mg ml^−1^ is shown in Fig. 5. Panel (a) shows that the vast majority of the molecules are isolated and can be observed anchored to the surface with different geometric orientations. Clusters of molecules are also visible through the sample (example in the purple square). Given the intrinsic 3D spatial character, a rich variety of orientations on the surface can be produced. The analysis of the size in isolated molecules, and their comparison with the expected values allow identifying their orientation over the gold substrate. Some of the most repeated ones are marked with colored circles, and their zoomed in images are classified with respect to their modelled orientation over the surface illustrated by a top view scheme. The measured sizes of the molecules agree with the models with a deviation of ± 0.01 nm. A third column represents the side view, *i.e.*, how the molecule is oriented with respect to the tip (triangular mark) and surface (brown surface).

**Fig. 6:**
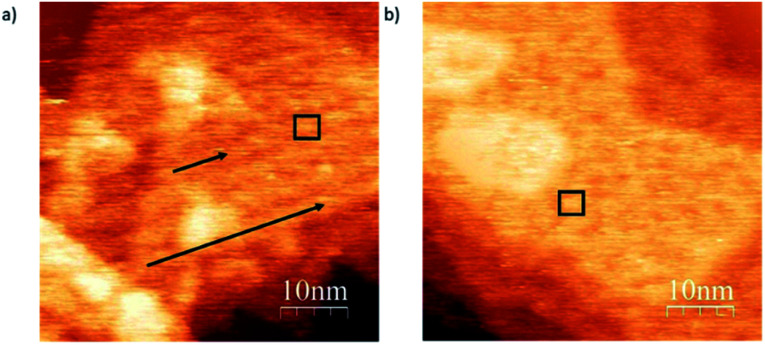
Topographic images of *exo*-dithia[7]helicene-1 and *endo*-dithia[7]helicene-2 molecules at room temperature in a concentration of 0.5 mg ml^−1^ deposited by drop casting and spin coating methods, respectively. (a) Topographic image of *exo* molecule clusters whose parameters are *I*_*t*_ = 1.21 nA and *V*_bias_ = −0.5 V. (b) Topographic image of *endo* clusters at *I*_*t*_ = 1.10 nA and *V*_bias_ = −0.7 V.

In order to show the role of the S atom and its binding interaction with the gold surface in deposition, we compare the structural differences of deposits for *exo* and *endo*-dithia[7]helicene respectively in a concentration of 0.5 mg ml^−1^ over large surface areas (Fig. 6). Panel (a) shows arranged self-assembly of the *exo*-dithia[7]helicene molecules over the terraces of Au (111) forming clusters with a width between 2.8 and 3 nm (see the black square). As the diameter of a single *exo*-dithia[7]helicene molecule is close to 0.75 nm (see Fig. S4f in the ESI†), every cluster shown in the panel is formed by an aggrupation of four molecules. It also seems that there is some molecular arrangement over the surface as black arrows indicate. The typical atomically flat terraces of reconstructed gold in the crystallographic direction (111) are reproduced for the molecular arrangement in Fig. 6a (see the triangular shape). The observation of these terraces means that an ordered layer of clusters is deposited over the gold. On the other hand, Fig. 6b shows a non-ordered disposition of *endo*-dithia[7]helicene molecular clusters around 2.8–4 nm. The diameter measured for the *endo* molecules is 0.73 ± 0.01 nm (close to the expected 0.72 nm and is illustrated in Fig. S4c in the ESI†). Following the previous reasoning, the clusters in this occasion would be formed by four or five molecules (see the square mark in Fig. 6b). In summary, it seems that for the case of thin layer deposition, there is a higher tendency to become ordered in the case of *exo*-dithia[7]helicene, likely resulting from a tighter binding with the surface, while for the *endo*-dithia[7]helicene there is a higher number of molecules per cluster, presumably as a result of an enlarged reticular energy as manifested in the solid.

### Synthetic procedures, materials and methods and crystallographic data

A full description of the synthetic methods, materials and scanning tunneling microscopy techniques used can be found in the ESI.†. Crystallographic data for 1 and 2 are available in the CIF format from the Cambridge Crystallographic Data Centre with the reference numbers CCDC 1974560 and 1974561, respectively.

## Conclusions

In order to access conductive coils minimized down to the molecular size and connectable to metallic electrodes, we have proposed maintaining the conjugated structure of a helicene while increasing the binding strength at the ends in contact by integrating sulfur atoms into the terminal rings without disrupting the conjugated nature of the molecule. Our target molecules were 3,14- and 1-16-dithia[7]helicene which display an *exo* and *endo* sulfur topology with respect to the helical structure, respectively (alternatively named *exo*-dithia[7]helicene-1 and *endo*-dithia[7]helicene-2). They could be efficiently synthesized through methods of modular assembly involving Sonogashira, hydroboration and Suzuki couplings followed by a Mallory photocyclization. Their crystal structures were resolved by X-ray diffraction corroborating the molecular structures, but also revealing important differences in their packing architectures, manifested in melting point differences of near 120 °C. *Exo*-1 and *endo*-2 displayed improved binding interactions and a conductive behavior between gold electrodes as could be inferred from STM imaging performed at room temperature. Imaging could be resolved down to one single molecule, exposing its electrical conductance and even allowing visual chiral assignment. With these results in hand we're currently planning to measure actual currents circulating between metallic tips anchored to the ends of these molecules, as well as to further coiled homologues that can be accessed through similar synthetic protocols. We expect a wealth of exciting electronic properties awaiting to be explored in the series of molecular solenoids that begin with *exo*-1 and *endo*-2.‡‡The contents of this work have been presented in the 2019 Conference on Materials & Nanomaterials (MNs-19), Jul. 17–29, 2019, Paris, France.

## Conflicts of interest

There are no conflicts to declare.

## Supplementary Material

NA-002-D0NA00045K-s001

NA-002-D0NA00045K-s002
