# Supplementary material for: Helical nanostructures for organic electronics: the role of topological sulfur in ad hoc synthesized dithia[7]helicenes studied in the solid state and on a gold surface
Source: Nanoscale Adv. 2020 Mar 16;2(5):1921–6. doi: 10.1039/d0na00045k (PMC9417725; doi:10.1039/d0na00045k)
Supplement: NA-002-D0NA00045K-s001 [file NA-002-D0NA00045K-s001.pdf]

Electronic Supplementary Material (ESI) for Nanoscale Advances.
This journal is © The Royal Society of Chemistry 2020

ELECTRONIC SUPPLEMENTARY INFORMATION

Helical Nanostructures for Organic Electronics: The Role of Topological Sulfur in Ad-Hoc Synthesized Dithia[7]helicenes Studied in the Solid and on a Gold Surface

Bianca C. Baciu,[1] Tamara de Ara,[2] Carlos Sabater,[2] Carlos Untiedt,[2] and Albert Guijarro*,[1]*

[1]Departamento de Química Orgánica and Instituto Universitario de Síntesis Orgánica, and

[2]Departamento de Física Aplicada, Campus de San Vicente del Raspeig, Universidad de Alicante, Apdo. 99, 03080 Alicante, Spain

E-mail: untiedt@ua.es, aguijarro@ua.es

Contents

Experimental Part 1

1. General Methods 1

2. Photochemistry 1

3. Synthesis and characterization of compounds 1

3.1. Diethyl (4-bromobenzyl) phosphonate 2

3.2. **(*E*)-2-(4-bromostyryl)thiophene** 2

3.3. **8-bromonaphtho[2,1-b]thiophene** 3

3.4. **8-ethynylnaphtho[2,1-b]thiophene** 4

3.5. **(*E*)-4,4,5,5-tetramethyl-2-(2-(naphtho[2,1-b]thiophen-8-yl)vinyl)-1,3,2-dioxoborolane** 5

3.6. **(*E*)-1,2-bis(naphtho[2,1-b]thiophene-8-yl)ethene** 6

3.7. ***Exo*-dithia[7]helicene-1** 6

4. ^{1}H NMR and ^{13}C NMR spectra of compounds 7

4.1. Diethyl (4-bromobenzyl)phosphonate (300MHz, $CDCl_3$) 8

4.2. (*E*)-2-(4-Bromostyryl)thiophene (300MHz, $CDCl_3$) 9

4.3. (*E*)-3-(4-Bromostyryl)thiophene (300MHz, $CDCl_3$) 10

4.4. 8-Bromonaphtho[2,1-b]thiophene (300MHz, $CDCl_3$) 11

4.5. 8-Bromonaphtho[1,2-b]thiophene (300MHz, $CDCl_3$) 12

4.6. 8-Ethynylnaphtho[2,1-b]thiophene (300MHz, $CDCl_3$) 13

4.7. 8-Ethynylnaphtho[1,2-b]thiophene (300MHz, $CDCl_3$) 14

4.8. (*E*)-4,4,5,5-Tetramethyl-2-(2-(naphtho[2,1-b]thiophen-8-yl)vinyl)-1,3,2-dioxoborolane (300MHz, $CDCl_3$) 15

4.9. (*E*)-4,4,5,5-tetramethyl-2-(2-(naphtho[1,2-b]thiophen-8-yl)vinyl)-1,3,2-dioxoborolane (300MHz, $CDCl_3$) 16

4.10. (*E*)-1,2-Bis(naphto[2,1-b]thiophene-8-yl)ethene (300MHz, $CDCl_3$) 17

4.11. (*E*)-1,2-Bis(naphto[1,2-b]thiophene-8-yl)ethene (300MHz, $CDCl_3$) 17

4.12. *Exo*-dithia[7]helicene-**1** (300MHz, $CDCl_3$) 18

4.13. *Endo*-dithia[7]helicene-**2** (300MHz, $CDCl_3$) 19

5. X-Ray diffraction (XRD) analysis 20

5.2 XRD Technical information 20

5.3 Geometrical details of the heterochiral molecular pair in the crystal 22

6. Differential scanning calorimetry (DSC) and melting point correction applied 23

7. HPLC analysis 25

8. Mass Spectroscopy (MS) 27

9. UV-vis Spectroscopy 28

10. Scanning Tunneling Microscopy (STM) 29

10.1. Procedure to deposit molecules over the substrate 29

10.2. SMT images of *exo*-dithia[7]helicene-**1** and *endo*-dithia[7]helicene-**2** with another concentration 29

10.3. Illustration of the geometries of *exo*-dithia[7]helicene-**1** and *endo*-dithia[7]helicene-**2** 30

Experimental Part

1. General Methods

Some chemicals and solvent were purchased from Aldrich, Fluorochem, TCI and Alfa Aesar Chemical Company as reagent grade and used without further purification unless otherwise stated. We dried and distilled some solvents like THF, benzene and cyclohexane over Na/K. The alloy was made before use. Commercially unavailable reagents were synthesized via different reaction like Sonogashira coupling, Suzuki coupling or others, but then we will explain each one separately.

Gas chromatography analyses (GLC) were carried out with a Hewlett Packard HP-5890 instrument equipped with a flame ionization detector and a 30 m HP-5 capillary column (0.32 mm diam, 0.25 μm film thickness), using nitrogen as carrier gas (12 psi). Column chromatography was performed with Merck silica gel 60 (0.040-0.063 μm, 240-400 mesh). Thin-layer chromatography (TLC) was performed on precoated silica gel plates (Merck 60, F254, 0.25 mm). TLC detection was done by UV_{254} light, R_f values are given under these conditions. NMR spectra were recorded on a Bruker Avance 300 and Bruker Avance 400 (300 and 400 MHz for ^{1}H-NMR, and 75 and 100 MHz for ^{13}C-NMR respectively) using $CDCl_3$ as a solvent and TMS as internal standard. Chemical shifts (δ) are given in ppm vs. TMS. Infrared (IR) analysis was performed with a JASCO FT/IR 4100 spectrophotometer equipped with an ATR component. LRMS were performed using the electron impact (EI) mode at 70 eV in an AGILENT 5973N mass spectrometer coupled with an AGILENT 6890N gas chromatographer. Melting points were performed with a Reichert Thermovar polarizing light microscope and melting points apparatus and have been corrected. Differential scanning calorimetry (DSC) analyses were performed with a calorimeter of TA INSTRUMENTS model Q100 mDSC. HPLC analysis was performed in an AGILENT 1100 Series chromatograph equipped with a C18 reversed phase 00G-4601-E0 Kinetex LC column. A double beam UV-vis spectrophotometer (Shimadzu UV-1603) was used for recording the electronic spectra.

2. Photochemistry

A 400-watt high-pressure mercury lamp (Osram HQL MBF-U) was modified by cutting away the outer glass envelope from the screw base (preserving the inner quartz arc tube containing Hg) and was mounted in a porcelain lamp holder provided with a reflector. The lamp was connected to a corresponding power unit and the light beam was focused to a number of 100mL Schlenck's tubes provided with magnetic stirring and a vertical condenser refligerated with a recirculating chiller (Huber MPC-K6) using a 30% ethylene glycol -water mixture as a coolant. The chemical hood was lined with aluminum foil to avoid unwanted exposure to UV radiation. We used cyclohexane or benzene under reflux as solvents.

3. Synthesis and characterization of compounds

The synthesis of fragments **3** and **4** was carried out according to the following scheme:

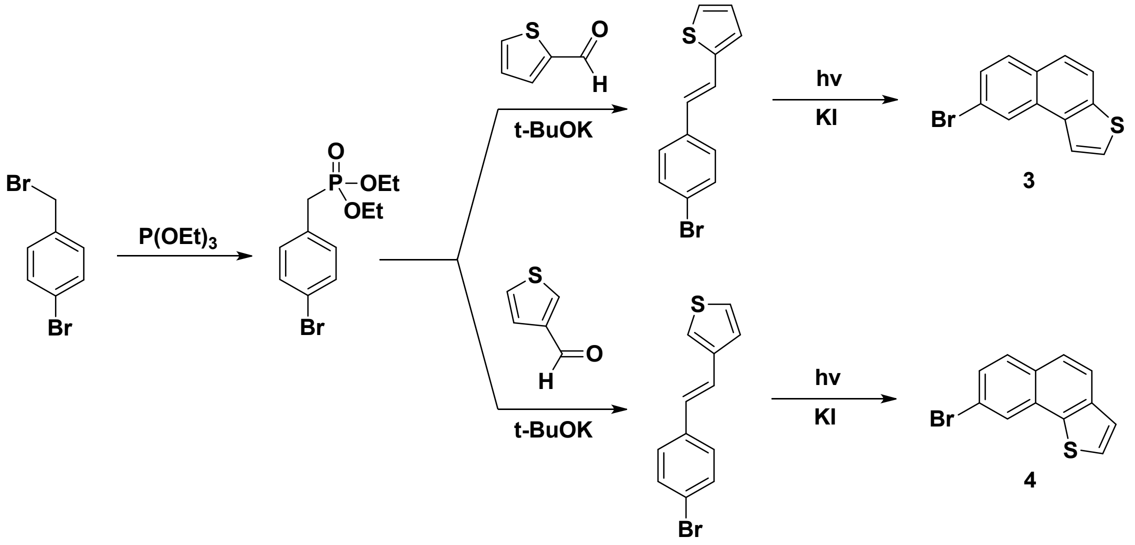

3.1. **Diethyl (4-bromobenzyl) phosphonate**[1]

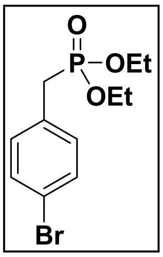

This compound was prepared by adapting the procedure described in the literature. In a 100mL round flask commercial 4-bromobenzyl (2.51 g; 10 mmol, 1eq.) bromide was heated in an oil bath under reflux (160ºC) in triethylphosphite (1.77mL; 10.2 mmol; 1.2 eq.) for 7 hours. After cooling to room temperature, the excess of triethylphosphite was removed under vacuum. A colorless oil was obtained in a near quantitative yield (95%).

Colorless oil; ^{1}H-NMR ($CDCl_3$, 300 MHz): *δ*= 7.41 (dd, *J*= 8.5, 2.5Hz, 2H), 7.15 (dd, *J*= 8.5,2.5Hz, 2H), 4.00 (m, 4H), 3.07 (d, *J*= 21.7Hz, 2H), 1.23 (t, *J*= 7.1Hz, 6H). ^{13}C-NMR ($CDCl_3$, 75 MHz): *δ*= 131.72 (d, *J*=3.0Hz, 2CH), 131.52 (d, *J*=6.6Hz, 2CH), 130.81 (d, *J*= 9.2Hz, 1C), 121,01 (d, *J*= 4.6Hz, 1CBr), 62.34 (d, *J*= 6.8Hz, $2CH_2O$), 33.32 (d, *J*= 138.6Hz, $1CH_2P$), 16.46 (d, *J*= 6.0Hz, $2CH_3$). MS (EI) *m/z* 308.00 (M^++2, 36.4), 306.05 (M^+ 36.3), 250.00 (22.5), 171.00 (99.0), 169.00 (100), 109.05 (62.4), 90.10 (42). IR (neat) ν_{max} 2982.37, 2908.13, 1359.48, 2341.16, 1487.81, 1237.11, 1050.05, 1022.09, 1012.45, 961.34, 853.35, 767.53, 728.96, 701.00 cm^{-1}.

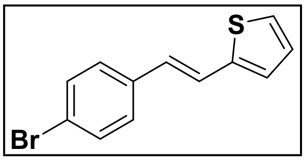

3.2. **(*E*)-2-(4-bromostyryl)thiophene**[2]

This compound was prepared by adapting the procedure described in the literature. In a 100mL round flask diethyl (4-bromobenzyl)phosphonate (0.6170 g; 2 mmol; 1eq.) was added, and three cycles of vacuum/argon were then performed. Then the flask was put in an ice bath and 2-thiophenecarboxaldheyde (0.280mL; 3 mmol; 1.5 eq.) was added via syringe. In other flask under argon a solution of KOt-Bu (0.8713 g; 7.5 mmol; 3.75 eq.) in 2mL of dry THF was prepared. When the bath temperature reached 0ºC the solution of KOt-Bu was added via syringe. Then 4 additional mL of dry THF were added and the mixture was stirred at room temperature for 3 hours. It was then extracted using 5mL of H_2O and 3x5mL of ethyl acetate (EtOAc). The solution was dried over magnesium sulfate, filtered and the solvent was evaporated under reduced pressure (15 Torr). The product was purified by column chromatography on silica gel (hexane) to afford a yellow solid in 90% purified yield. Calculated yield by 300 MHz ^{1}H-NMR using an internal standard (99% 1,3,5-trimethoxibenzene) was 96 %.

Yellow solid; melting point (corrected) 124.1ºC; R_f= 0.47 (hexane); ^{1}H-NMR ($CDCl_3$, 300 MHz): δ= 7.49-7.40 (m, 2H), 7.34-7.26 (m, 2H), 7.24-7.15 (m, 2H), 7.09-6.96 (m, 2H), 6.84 (d, J= 16.1Hz, 1H). ^{13}C-NMR ($CDCl_3$, 75 MHz): δ= 142.59 (1C), 136.04 (1C), 131.92 (2CH), 127.85 (2CH), 127.81 (1CH), 127.08 (1CH), 126.65 (1CH), 124.86 (1=CH), 122.59 (1=CH), 121.36 (1CBr). MS (EI) *m/z* 266.0 (M^++2, 93.5), 264.0 (M^+, 93.8), 184.10 (100), 185.10 (41.5), 152.10 (39.8), 92.10 (27.6). IR (neat) ν_{max} 3077.83, 3011.80, 1619.91, 1438.96, 1267.00, 1225.54, 1071.26, 1004.73, 957.48, 945.91, 828.27, 804.17, 697.14, 665.32 cm^{-1}.

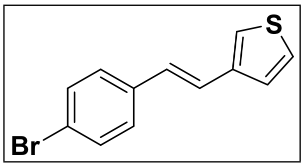

(*E*)-3-(4-bromostyryl)thiophene[3]

This compound was prepared by the same procedure as (*E*)-2-(4-bromostyryl)thiophene but using 3-thiophenecarbox-aldehyde as starting reagent in 79% isolated yield. Calculated yield by 300 MHz ^{1}H-NMR using an internal standard (99% 1,3,5-trimethoxibenzene) was 87 %.

White solid; melting point (corrected) 136.5ºC; R_f= 0.37 (hexane); ^{1}H-NMR ($CDCl_3$, 300 MHz): δ= 7.49-7.41 (m, 2H), 7.37-7.21 (m, 5H), 7.10 (d, J= 16.3Hz, 1H), 6.86 (d, J= 16.3Hz, 1H). ^{13}C-NMR ($CDCl_3$, 75 MHz): δ= 139.88 (1C), 136.45 (1C), 131.89 (2CH), 127.87 (2CH), 127.47 (1CH), 126.50 (1CH), 124.93 (1CH), 123.69 (1CH), 122.98 (1CH), 121.22 (1CBr). MS (EI) *m/z* 266.0 (M^++2, 45.2), 264.0 (M^+, 43.7), 184.1 (100), 185.1 (76.8), 152.1 (21.6), 92.2 (20.8). IR (neat) ν_{max} 3091.33, 3004.55, 1627.63, 1581.34, 1482.99, 1397.17, 1239.04, 1204.33, 1102.12, 1070.30, 1005.70, 967.12, 863.95, 848.52, 771.38, 704.85 cm^{-1}.

3.3. **8-bromonaphtho[2,1-b]thiophene**[2]

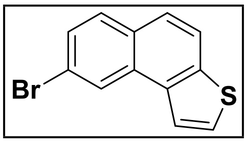

This compound was prepared using the main photochemical setup described above. A solution/suspension of (*E*)-2-(4-bromostyryl)thiophene (26.5 mg; 0.1 mmol; 1eq.) and KI (16.6 mg; 0.1 mmol; 1eq.) in cyclohexane (100mL) was prepared in a Schlenck tube provided with a vertical condenser connected to a chiller. The recirculating chiller was turned on and the mixture was irradiated with a 400 W high-pressure Hg lamp for 3-4 hours under reflux. The advance of the reaction was followed by GS. After the reaction was completed, it was washed with aqueous $NaHSO_3$, dried over magnesium sulfate, filtered and the solvent evaporated under reduced pressure (15 Torr). The product was purified by recrystallization from EtOAc to obtain a white solid. We calculated a 90% yield using an internal standard (1,3,5-trimethoxibenzene (99%).

White solid; melting point (corrected) 110.1ºC (EtOAc) (lit. 142-144ºC)[2]; R_f= 0.44 (hexane); ^{1}H-NMR ($CDCl_3$, 300 MHz): δ= 8.46 (d, J= 1.9Hz, 1H), 7.93-7.85 (m, 2H), 7.79 (d, J= 8.6Hz, 1H), 7.67 (d, J= 8.8Hz, 1H), 7.63-7.57 (m, 2H). ^{13}C-NMR ($CDCl_3$, 75 MHz): δ= 138.32 (1C), 135.03 (1C), 130.53 (1C), 130.23 (1CH), 129.51 (1C), 128.67 (1CH), 126.48 (1CH), 126.25 (1CH), 124.68 (1CH), 121.92 (1CH), 121.23 (1CH), 120.63 (1CBr). MS (EI) *m/z* 264.0 (M^++2, 100), 262.0 (M^+, 95.5), 183.1 (26.6), 139.1 (40.3), 91.2 (20.5). IR (neat) ν_{max} 1603.52, 1577.49, 1500.35, 1466.60, 1371.14, 1157.08, 1143.58, 1070.30, 921.80, 874.56, 652.78 cm^{-1}.

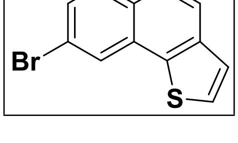

8-bromonaphtho[1,2-b]thiophene

Following the previous procedure, a solution of (*E*)-3-(4-bromostyryl)thiophene was irradiated using a 400W high pressure Hg lamp. We calculated a 83% yield using an internal standard (99% 1,3,5-trimethoxibenzene).

White solid; melting point 89.2ºC (EtOAc); R_f=0.6 (hexane); ^{1}H-NMR ($CDCl_3$, 300 MHz): δ= 8.29 (d, *J*= 1.9Hz, 1H), 7.82 (d, *J*= 8.6 Hz, 1H); 7.78 (d, *J*= 8.7Hz, 1H), 7.67 (d, *J*= 8.4Hz, 1H), 7.58 (dd, *J*= 8.7, 1.9Hz, 1H), 7.55 (d, *J*= 5.3Hz,1H), 7.46 (d, *J*= 5.3Hz, 1H). ^{13}C-NMR ($CDCl_3$, 75 MHz): δ= 138.17 (1C), 136.33 (1C), 130.54 (1CH), 130.34 (1C), 129.35 (1C), 128.95 (1CH), 126.06 (1CH), 126.04 (1CH), 125.33 (1CH), 125.08 (1CH), 122.60 (1CH), 120.70 (1CBr). MS (EI) *m/z* 264.0 (M^++2, 100), 262.0 (M+, 95.9), 183.1 (29.6), 139.1 (46.3), 91.1 (18.2). IR (neat) ν_{max} 1607.38, 1506.13, 1472.38, 1290.14, 1252.54, 1068.37, 980.62, 868.77, 763.67, 699.06 cm^{-1}.

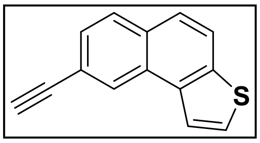

3.4. 8-ethynylnaphtho[2,1-b]thiophene

This compound was prepared by adapting a Sonogashira coupling described in the literature to our substrates.[4] A pressure tube was charged with a stirbar, $Pd(PPh_3)_2Cl_2$ (70.716 mg; 0.1 mmol; 0.1 eq.), CuI (21.450 mg; 0.1 mmol; 0.1eq.), 8-bromonaphtho[2,1-b]thiophene (248.37 mg; 1 mmol; 1 eq.), sealed with septum and three cycles vacuum/argon were then performed. Then, 2.2mL of dry THF and 1.1mL of dry piperidine followed by TMS-acetylene (282.64 µL; 2 mmol; 2 eq.) were added. The tube was closed and heated in an oil bath at 120ºC overnight. The reaction was monitired by TLC. After completion, the reaction was filtered through a pad of celite. The crude product was evaporated onto silica gel and purified by column chromatography (hexane). The combined fractions were evaporated, and the product was immediately dissolved in MeOH (10 ml) and treated with solid K_2CO_3 (250 mg) with vigorous stirring for 3 hours. The work up consisted in an extraction using 5mL of H_2O and 3x10mL of EtOAc. The solution was then dried over magnesium sulfate, filtered and the solvent evaporated under reduced pressure (15 Torr). The residue was purified by column chromatography on silica gel (hexane) to obtain a yellow solid in 63% yield.

Yellow solid; melting point 94.7ºC (corrected); R_f=0.40 (hexane); ^{1}H-NMR ($CDCl_3$, 300 MHz): δ= 8.52-8.49 (m, 1H), 7.95 (dd, *J*= 5.4, 0.8Hz, 1H), 7.92-7.84 (m, 2H), 7.70 (d, *J*= 8.8Hz, 1H), 7.64-7.56 (m, 2H), 3.23 (s, 1H). ^{13}C-NMR ($CDCl_3$, 75 MHz): δ= 138.08 (1C), 135.62 (1C), 130.82 (1C), 128.89 (1C), 128.69 (1CH), 128.25 (1CH), 128.08 (1CH), 126.56 (1CH), 124.72 (1CH), 122.02 (1CH), 121.82 (1CH), 119.93 (1C), 84.35 (1C) 77.86 (1CH). MS (EI) *m/z* 208.10 (100), 209.10 (16), 164.10 (8.55), 163.10 (20). IR (neat) ν_{max} 3272.61, 2935.13, 1593.88, 1470.46, 1372.10, 1205.29, 1192.76, 1150.33, 886.13, 709.67, 662.42 cm^{-1}.

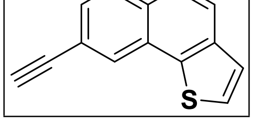

8-ethynylnaphtho[1,2-b]thiophene

This compound was prepared employing the same procedure as in the case of 8-ethynylnaphto[2,1-b]thiophene, by replacing 8-bromonaphto[2,1-b]thiophene by 8-bromonaphto[1,2-b]thiophene.

White solid in 82% yield; melting point 54.2ºC (corrected); R_f=0.36 (hexane); ^{1}H-NMR ($CDCl_3$, 300 MHz): δ= 8.33 (s, 1H), 7.87 (d, *J*= 8.4 Hz, 1H); 7.84 (d, *J*= 8.7 Hz, 1H), 7.69 (d, *J*= 8.6Hz, 1H), 7.58 (dd, *J*= 8.4, 1.6Hz, 1H), 7.54 (d, *J*= 5.3Hz, 1H), 7.46 (d, *J*=

5.3Hz, 1H), 3.23 (s, 1H). ^{13}C-NMR ($CDCl_3$, 75 MHz): δ= 138.00 (HetArC, 1C), 137.15 (HetArC, 1C), 130.64 (HetArC, 1C), 129.01 (HetArC-H, 1C), 128.71 (HetArC, 1C), 128.52 (HetArC-H, 1C), 127.93 (HetArC-H, 1C), 125.78 (HetArC-H, 1C), 125.18 (HetArC-H, 1C), 125.08 (HetArC-H, 1C), 123.18 (HetArC-H, 1C), 120.15 (HetAr*C*-C≡, 1C), 84.01 (*C*≡CH, 1C), 78.16 (C≡*C*-H, 1C). MS (EI) *m/z* 208.10 (100), 209.10 (16.3), 164.10 (6.20), 163.10 (17.2). IR (neat) ν_{max} 3304.43, 2924.52, 2103.96, 1402.00, 1346.07, 1260.25, 1089.58, 999.91, 888.06, 774.23, 704.85, 661.46 cm^{-1}.

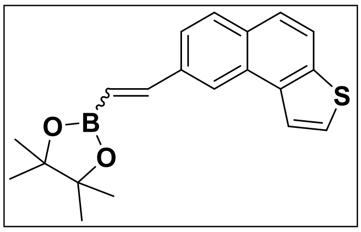

3.5. (*E*)-4,4,5,5-tetramethyl-2-(2-(naphtho[2,1-b]thiophen-8-yl)vinyl)-1,3,2-dioxoborolane

The compound was prepared by adapting to our substrates a procedure of hydroboration of alkynes.[5] In an oven-dried Schlenk tube, CuCl (3.66 mg, 0,015 mmol, 0.03 eq.), NaOt-Bu (3.45 mg, 0.030 mmol, 0.06 eq.) and Xantphos ligand (8.76 mg, 0.015 mmol, 0.03 eq.) was added. Then, after three cycles of vacuum/argon, 4.5mL of dry THF were injected. The solution was stirred for 30 min at room temperature and then, bis(pinacolato)diboron (271 mg, 1.05 mmol, 2 eq.) in 3mL of dry THF were added. The reaction mixture was stirred for 10 min and 8-ethinylnaphto[2,1-b]thiophene (109.2 mg, 0,525 mmol, 1eq.), was added followed by MeOH (40 µL, 1mmol). The reaction mixture was stirred at room temperature until no starting material was detected by TLC (3 hours). After 3 hours the reaction mixture was filtered through a pad of celite and the residue was purified by column chromatography on silica gel (hexane/toluene 98.5:1.5) obtaining a colorless oil in 76% yield.

Colorless oil; R_f=0.33 (hexane/toluene 9.85:0.15); ^{1}H-NMR ($CDCl_3$, 300 MHz): δ= 8.40-8.31 (m, 1H), 7.95 (dd, *J*= 5.4, 0.7Hz, 1H), 7.92-7.84 (m, 2H), 7.74 (dd, J=8.5, 1.6Hz, 1H), 7.72-7.59 (m, 3H), 6.37 (d, *J*= 18.4Hz, 1H), 1,36 (s, 12H). ^{13}C-NMR ($CDCl_3$, 75 MHz): δ= 149.77, 137.86, 136.28, 135.59, 131.40, 129.43, 128.95, 126.28, 124.82, 123.72, 123.03, 121.96, 121.24, 83.55 (PinC, 2C), 24.98 ($PinCH_3$, 4C). MS (EI) *m/z* 338.15 (M^++2, 7.0), 337.20 (M^++1, 22.8), 336.20 (M^+, 100), 335.20 (24.8), 251.15 (8.0), 236.10 (44.4), 220.10 (52.2), 184.10 (9.2), 139.15 (5.5), 57.15 (7.4). IR (neat) ν_{max} 2976.59, 2928.38, 2360.44, 1616.06, 1371.14, 1354.75, 1323.89, 1108.87, 969.05, 843.70, 825.38, 713.53, 672.07 cm^{-1}.

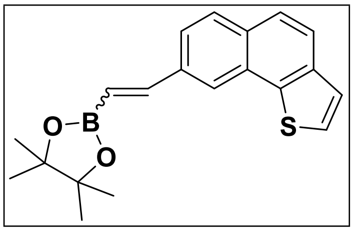

(*E*)-4,4,5,5-tetramethyl-2-(2-(naphtho[1,2-b]thiophen-8-yl)vinyl)-1,3,2-dioxoborolane

This compound was prepared following the previous procedure, replacing the terminal alkyne by 8-ethynylnaphto[1,2-b]thiophene.

White solid in 53% yield; melting point 137.5ºC (corrected); R_f=0.58 (hexane/toluene 9.5:0.5); ^{1}H-NMR ($CDCl_3$, 300 MHz)): δ= 8.21-8.15 (m, 1H), 7.88 (d, *J*= 8.5Hz, 1H), 7.81 (d, *J*= 8.6Hz, 1H), 7.75-7.58 (m, 3H), 7.52 (d, *J*= 5.3Hz, 1H), 7.45 (d, *J*= 5.3Hz, 1H), 6.36 (d, *J*= 18.4Hz, 1H), 1.35 (s, 12H). ^{13}C-NMR ($CDCl_3$, 75 MHz): δ= 149.42, 137.84, 135.81, 131.21, 129.24, 129.20, 125.41, 125.22, 125.12, 123.60, 123.40, 122.63, 83.58 (PinC, 2C), 24.99 ($PinCH_3$, 4C). MS (EI) *m/z* 338.15 (M^++2, 6.5), 337.25 (M^++1,

25.2), 336.20 (M^+, 100), 335.20 (23.7), 281.10 (6.0), 236.15 (51.6), 220.10 (64.2), 209.10 (19.3), 163.05 (4.0), 57.15 (8.9). IR (neat) v_{max} 2974.66, 2922.59, 1614.13, 1360.53, 1334.50, 1321.96, 1136.83, 995.10, 847.56, 832.13, 713.53, 670.14 cm^{-1}.

3.6. (*E*)-1,2-bis(naphtho[2,1-b]thiophene-8-yl)ethene

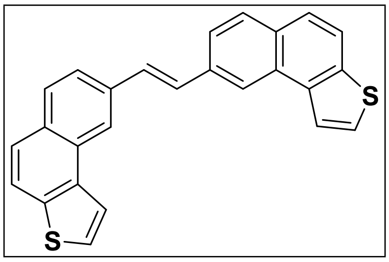

This compound was prepared by adapting to our substrates a Suzuki coupling described in the literature.[6] To an oven-dried pressure tube, $PdCl_2$ (9.64 mg, 0.03 mmol, 0.10 eq.), PPh_3 (15.78 mg, 0.07 mmol, 0.20 eq.), Cs_2CO_3 (269.22 mg, 0.9 mmol, 3 eq.), (*E*)-4,4,5,5-tetramethyl-2-(2-(naphtho[2,1-b]thiophen-8-yl)vinyl)-1,3,2-dioxoborolane (125.9 mg, 0.3 mmol, 1 eq.) and 8-bromonaphto[2,1-b]tiohene (81.13 mg, 0.3 mmol, 1 eq.) were added. The tube was sealed with a septum and after three cycles of vacuum/argon, 1.8mL of THF and 0.2mL of H_2O were added with a syringe. The tube was closed and heated in an oil bath at 85ºC for 20 hours. After the reaction was completed, it was extracted using 3mL of H_2O and 3x5mL of CH_2Cl_2. A rather insoluble solid in suspension was observed in the organic phase. The product, a greenish yellow solid, was separated by filtration. On the other hand, the combined extracts were solvent-evaporated and purified by column chromatography on silica gel (hexane/ethyl acetate 9:1) to obtain an additional fraction of the same green-yellow solid which was incorporated to the formerly filtered solid. A combined yield of 40% was accounted for.

Greenish yellow solid; melting point 311.9ºC (corrected); R_f=0.39 (hexane/AcOEt 9:1); ^{1}H-NMR ($CDCl_3$, 300 MHz): δ= 8.47-8.42 (m, 2H), 8.08 (dd, *J*= 5.4, 0.7Hz, 2H), 7.97 (d, *J*= 8.5Hz, 2H), 7.91-7.85 (m, 4H), 7.75 (d, *J*= 8.8Hz, 2H), 7.64 (d, *J*= 5.1Hz, 2H), 7.56 (s, 2H). MS (EI, DIP) *m/z* 394.0 (M^++2, 12.02), 393.1 (M^++1, 32.35), 392.0 (M^+, 100), 195.8 (16.06), 173.9 (9.36), 57.1 (4.78), 44.0 (43.30). IR (neat) v_{max} 1508.06, 1372.10, 1189.86, 1152.26, 1089.58, 960.37, 880.34, 827.31, 712.57, 665.32 cm^{-1}.

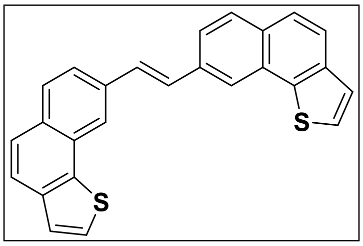

(*E*)-1,2-bis(naphto[1,2-b]thiophene-8-yl)ethene

This compound was prepared following the previous procedure, but using (*E*)-4,4,5,5-tetramethyl-2-(2-(naphtho[1,2-b]thiophen-8-yl)vinyl)-1,3,2-dioxoborolane and 8-bromonaphto[1,2-b]thiophene as starting reagents.

Greenish yellow solid in 56.4% yield; melting point 267,19ºC (corrected); R_f=0.22 (hexane/AcOEt 9:1); ^{1}H-NMR ($CDCl_3$, 300 MHz): δ= 8.26-8.22 (m, 2H), 7.95 (d, *J*= 8.5Hz, 2H), 7.89-7.79 (m, 4H), 7.73 (d, *J*= 8.6Hz, 2H), 7.56 (d, *J*= 5.3 Hz, 2H), 7.52 (s, 2H), 7.48 (d, *J*= 5.3 Hz, 2H). MS (EI, DIP) *m/z* 394.0 (M^++2, 12.02), 393.1 (M^++1, 32.35), 392.0 (M^+, 100), 195.8 (16.06), 173.9 (9.36), 57.1 (4.78), 44.0 (43.30). IR (neat) v_{max} 1716.34, 1540.85, 1508.06, 1318.11, 1267.00, 1087.66, 961.34, 880.34, 830.20, 797.45, 774.28, 712.57, 667.25 cm^{-1}.

3.7. *Exo*-dithia[7]helicene-1[7]

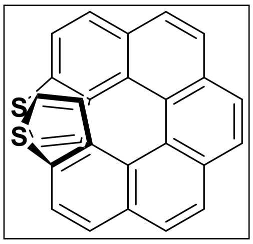

(E)-1,2-Bis(naphto[2,1-b]thiophene-8-yl)ethene (47.88 mg, 0.12 mmol, 1eq.) and KI (19.92 mg, 0,12 mmol, 1 eq.) were mixed in 200mL of benzene and placed in two oven-dried Schlenk tubes provided with vertical condensers connected to a chiller. The recirculating chiller was turned on and the mixture was irradiated with a 400 W high-pressure Hg lamp for 3-4 hours under reflux. The advance of the reaction was followed by TLC. After the reaction was completed, it was washed with aqueous $NaHSO_3$, dried over magnesium sulfate, filtered and the solvent evaporated under reduced pressure (15 Torr). The residue was purified by column chromatography on silica gel (hexane/ethyl acetate 9:1) to obtain a light brown solid. Recrystallization from EtOAc afforded light brown crystals in 52% yield.

Light brown crystals; melting point (DSC) 231.1ºC (EtOAc) (lit. 229-231ºC)[7]; R_f=0.48 (hexane/ AcOEt 9;1); ^{1}H-NMR ($CDCl_3$, 300 MHz): δ= 8.10-8.04 (m, 4H), 7.98 (d, J= 8.4Hz, 2H), 7.92-7.84 (m, 4H), 6.60 (d, J= 5.6Hz, 2H), 6.24 (dd, J= 5.6, 0.5Hz, 2H). ^{13}C-NMR ($CDCl_3$, 75 MHz): δ= 138.55 (2C), 135.34 (2C), 132.26 (2C), 129.91 (2C), 127.94 (2CH), 127.87 (2C), 127.08 (2CH), 125.43 (2CH), 125.14 (2C), 124.64 (2CH), 124.16 (2CH), 123.00 (2CH), 121.42 (2CH). MS (EI, DIP), m/z 392.1 (M^++2, 13.30), 391.1 (M^++1, 29.62), 390.1 (M^+, 100), 356.1 (29.07), 343.1 (37.00), 178.1 (23.60). IR (neat) ν_{max} 1343.18, 1313.29, 1271.82, 1193.72, 1158.04, 1149.37, 839.85, 801.28, 760.78, 716.42, 660.50 cm^{-1}.

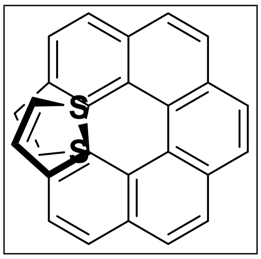

***Endo*-dithia[7]helicene-2**

In this case a solution of (E)-1,2-bis(naphto[1,2-b]thiophene-8-yl)ethene in benzene was irradiated following the same overall methodology described above for the *exo* isomer. Recrystallization from EtOAc afforded yellow crystals in 86% yield

Yellow crystals; melting point (DSC) 350.3ºC (EtOAc); R_f=0.35 (hexane/AcOEt 9:1); ^{1}H-NMR ($CDCl_3$, 300 MHz): δ= 8.18 (d, J= 8.4Hz, 2H), 8.07-7.99 (m, 6H), 7.91 (d, J= 8.4Hz, 2H), 7.06 (d, J= 5.4Hz, 2H), 6.80 (d, J= 5.4Hz, 2H). ^{13}C-NMR ($CDCl_3$, 75 MHz): δ= 138.23 (2C), 136.50 (2C), 132.76 (2C), 130.59 (2C), 128.80 (2CH), 128.26 (2C), 126.81 (2CH), 125.61 (2CH), 125.16 (2CH), 124.10 (2CH), 123.05 (2C), 122.84 (2CH), 122.59 (2CH). MS (EI, DIP), m/z 392.1 (M^++2, 17.54), 391.1 (M^++1, 29.82), 390.1 (M^+, 100), 356.1 (26.25), 343.1 (11.42), 313.1 (14.56), 300.1 (71.27). IR (neat) ν_{max} 1367.28, 1349.93, 1276.65, 1099.23, 1072.23, 840.81, 820.56, 787.78, 715.46, 703.89, 688.46, 660.50 cm^{-1}.

4. ^{1}H NMR and ^{13}C NMR spectra of compounds

4.1. Diethyl (4-bromobenzyl)phosphonate (300MHz, $CDCl_3$)

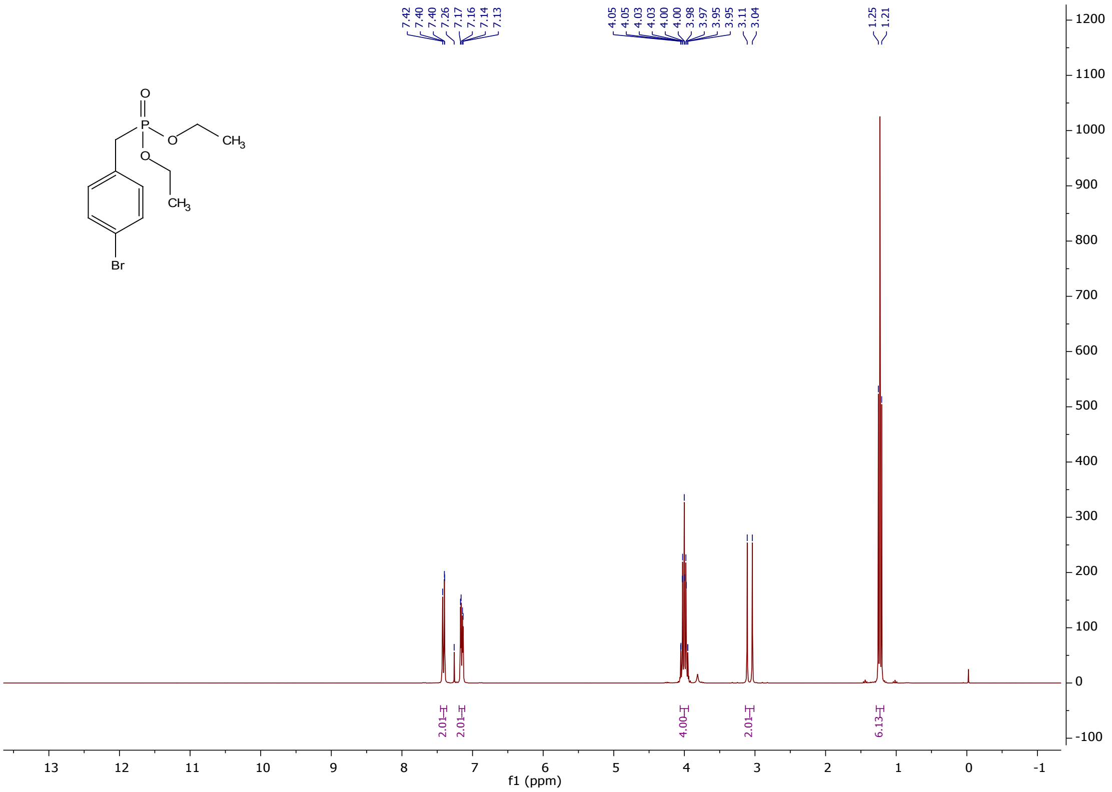

Diethyl (4-bromobenzyl)phosphonate (75MHz, $CDCl_3$)

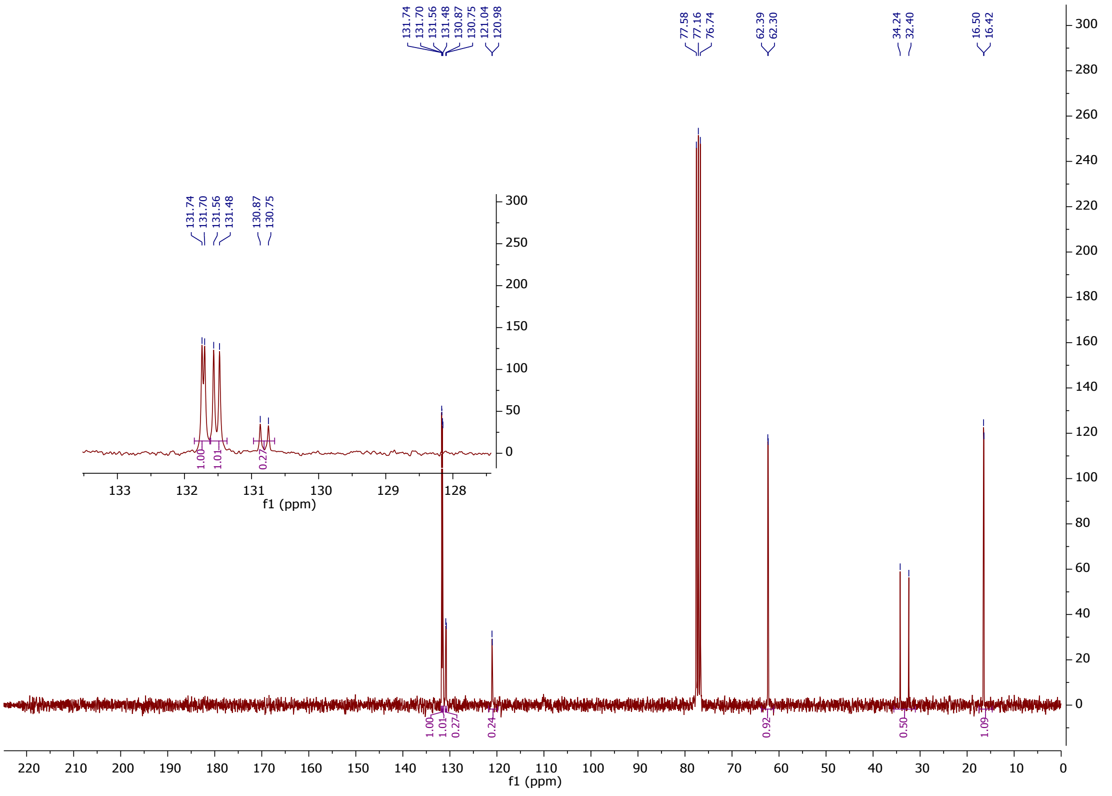

4.2. (*E*)-2-(4-Bromostyryl)thiophene (300MHz, $CDCl_3$)

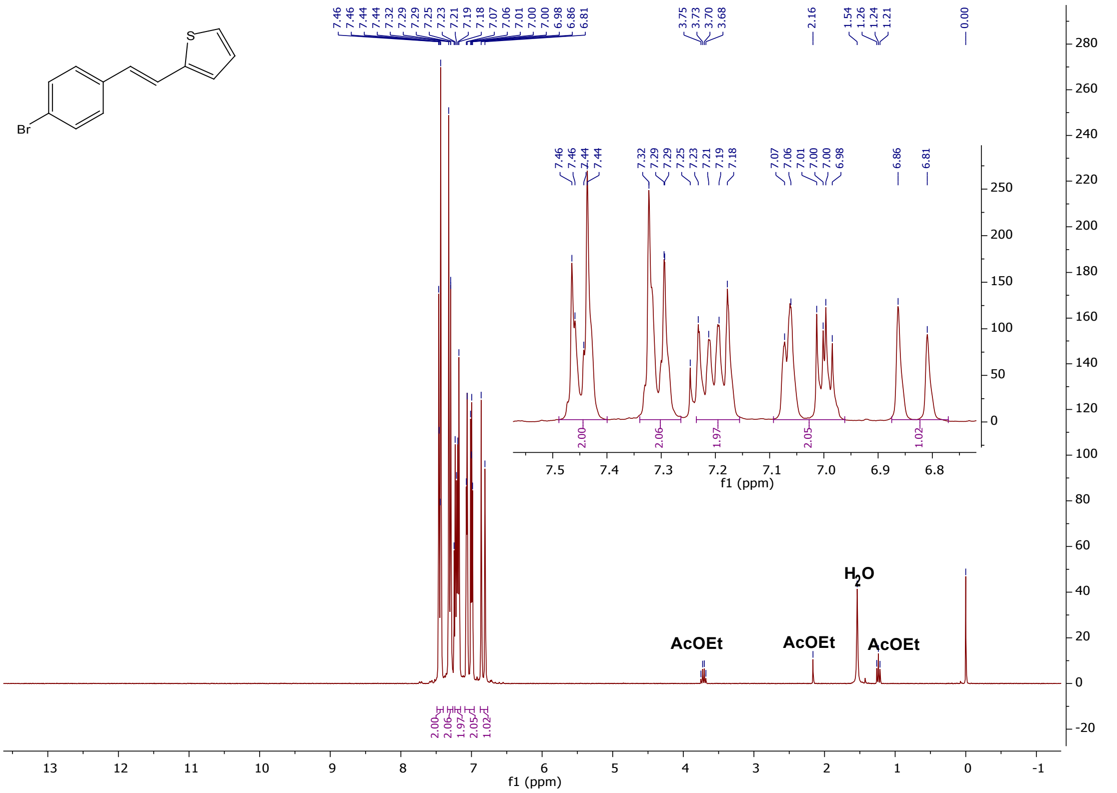

(*E*)-2-(4-Bromostyryl)thiophene (75MHz, $CDCl_3$)

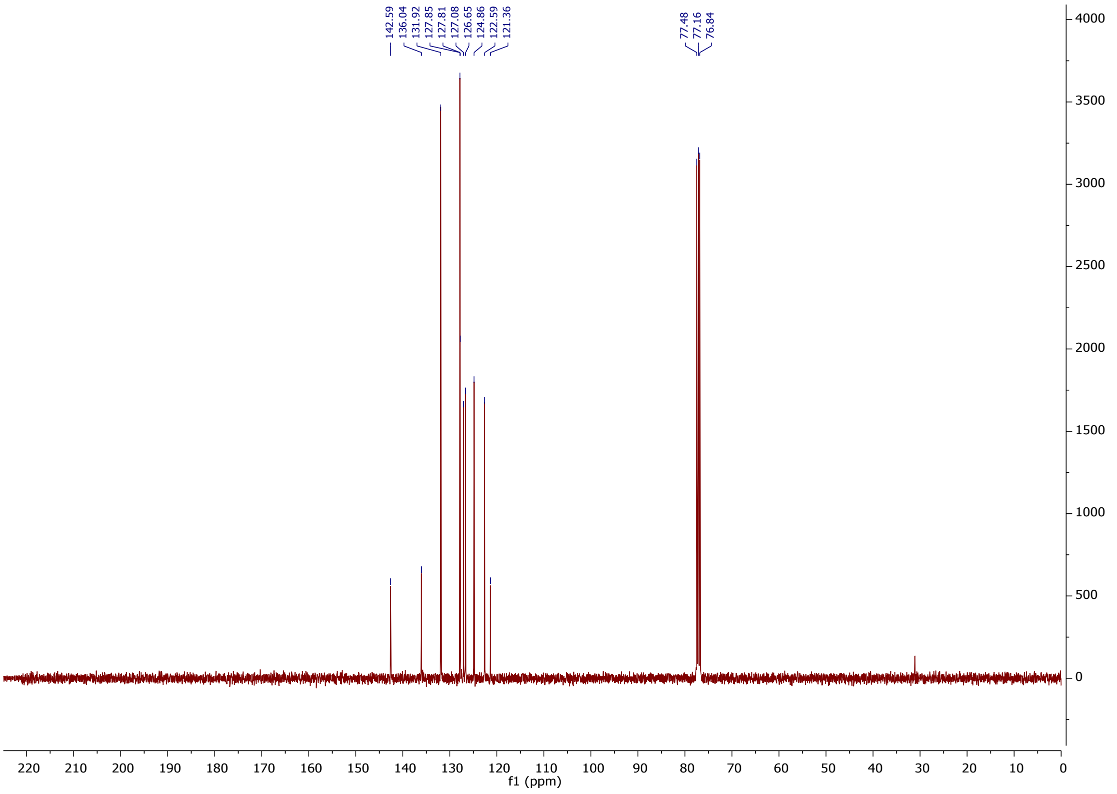

4.3. (*E*)-3-(4-Bromostyryl)thiophene (300MHz, $CDCl_3$)

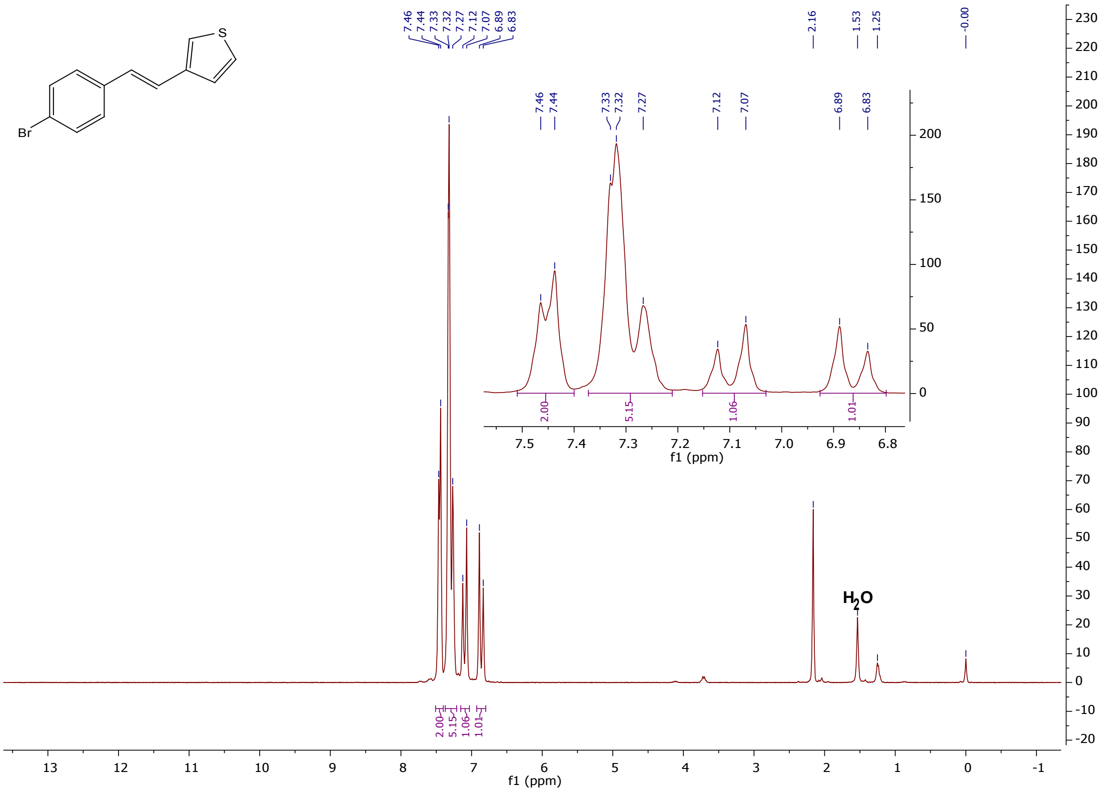

(*E*)-3-(4-Bromostyryl)thiophene (75MHz, $CDCl_3$)

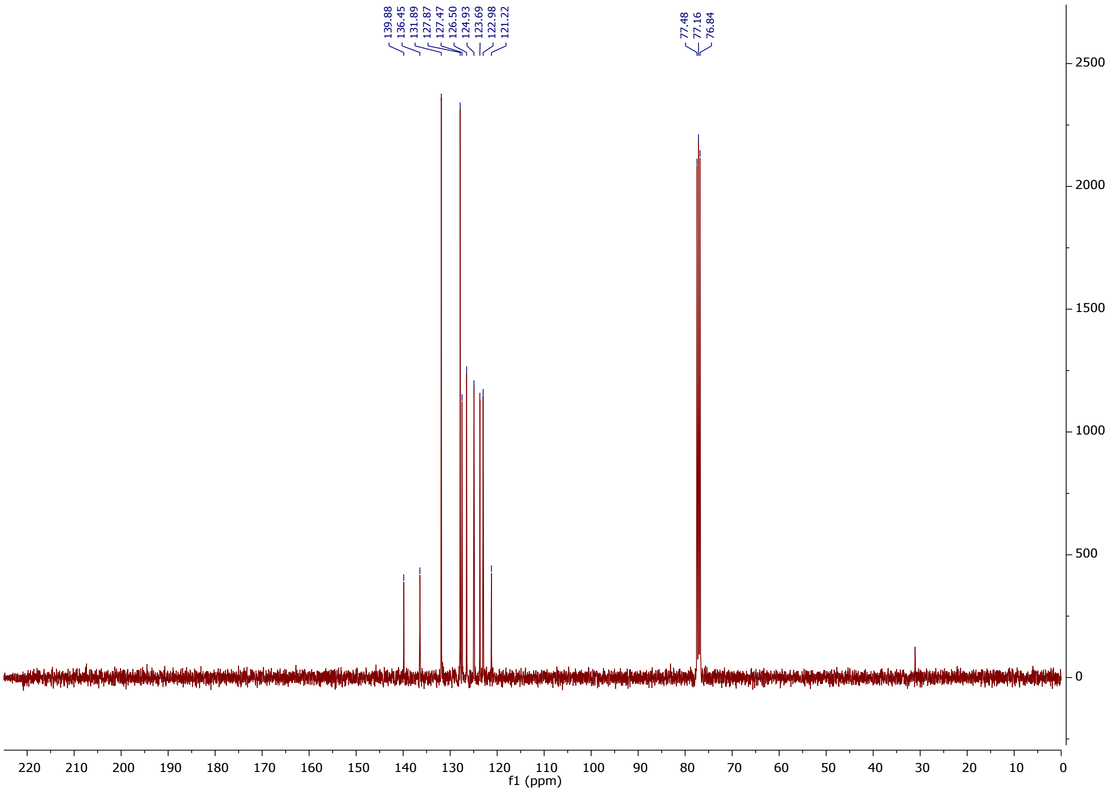

4.4. 8-Bromonaphtho[2,1-b]thiophene (300MHz, $CDCl_3$)

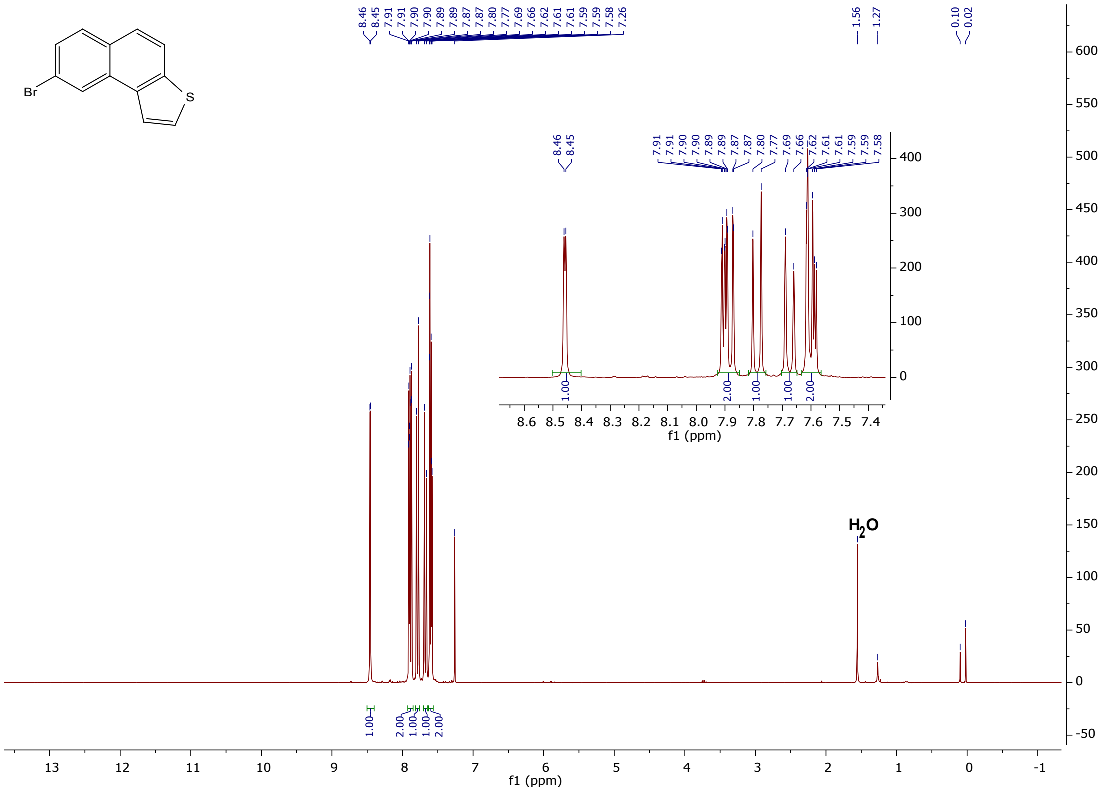

8-Bromonaphtho[2,1.b]thiophene (75MHz, $CDCl_3$

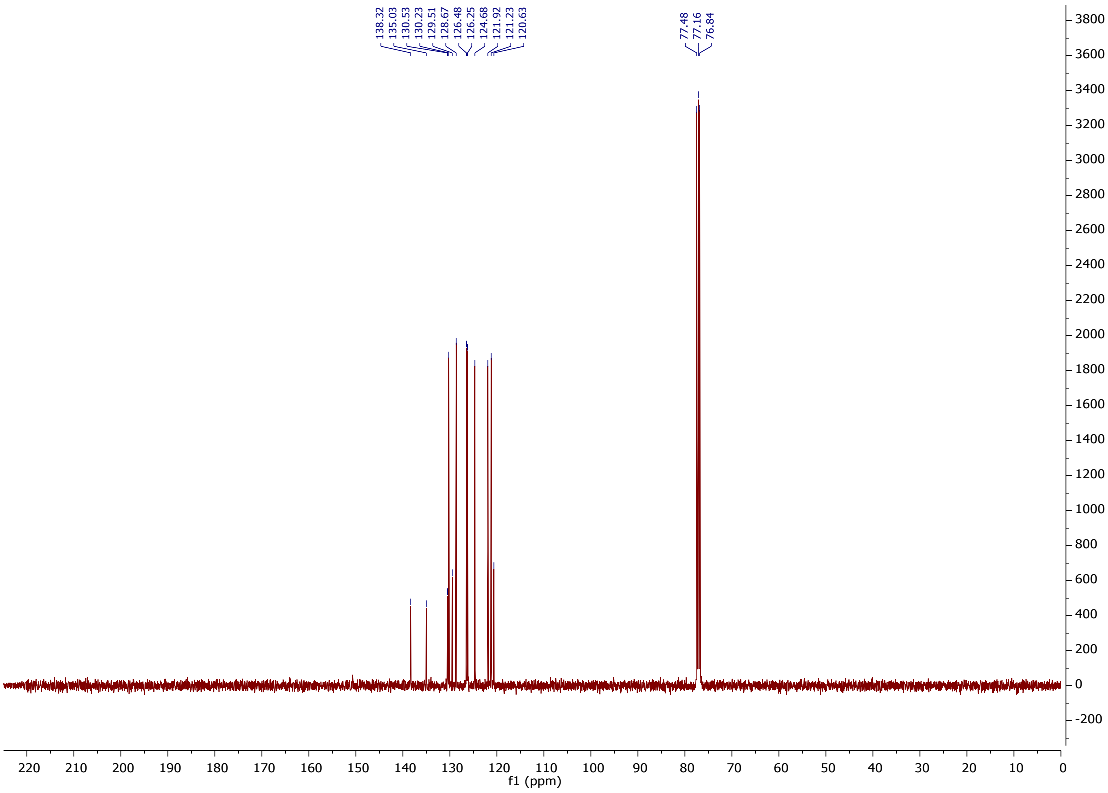

4.5. 8-Bromonaphtho[1,2-b]thiophene (300MHz, $CDCl_3$)

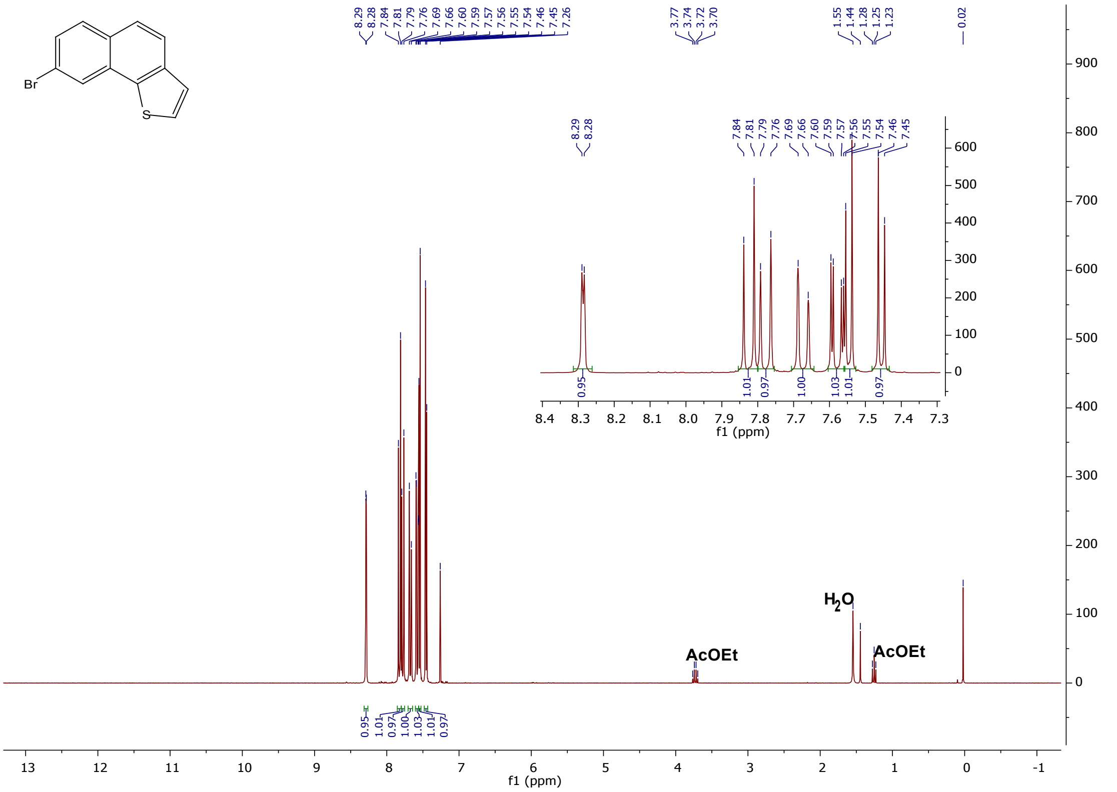

8-Bromonaphtho[1,2-b]thiophene (75MHz, $CDCl_3$)

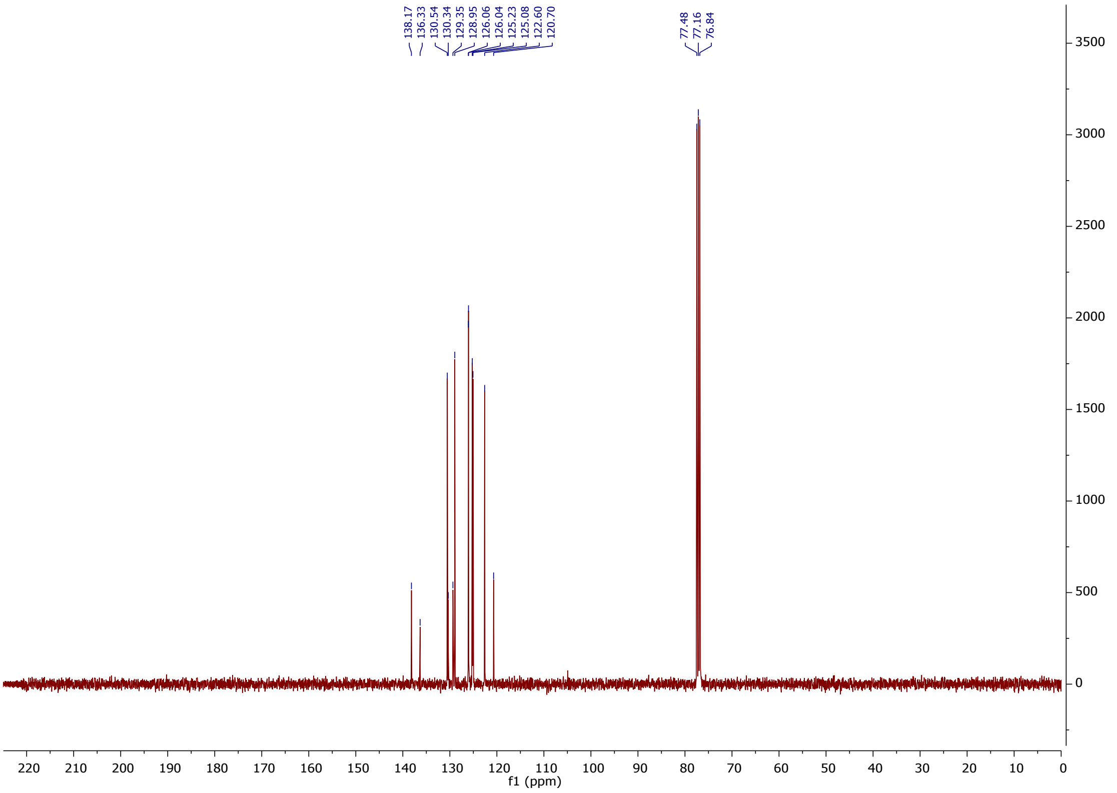

4.6. 8-Ethynylnaphtho[2,1-b]thiophene (300MHz, $CDCl_3$)

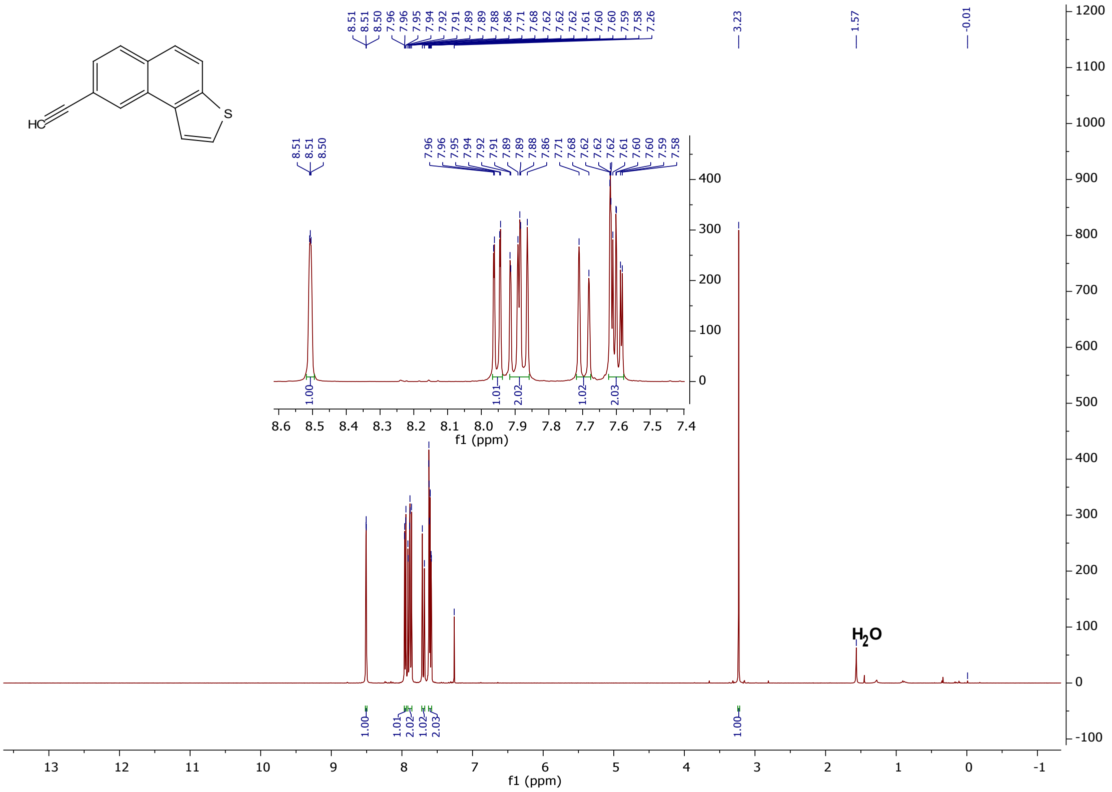

8-Ethynylnaphtho[2,1-b]thiophene (75MHz, $CDCl_3$)

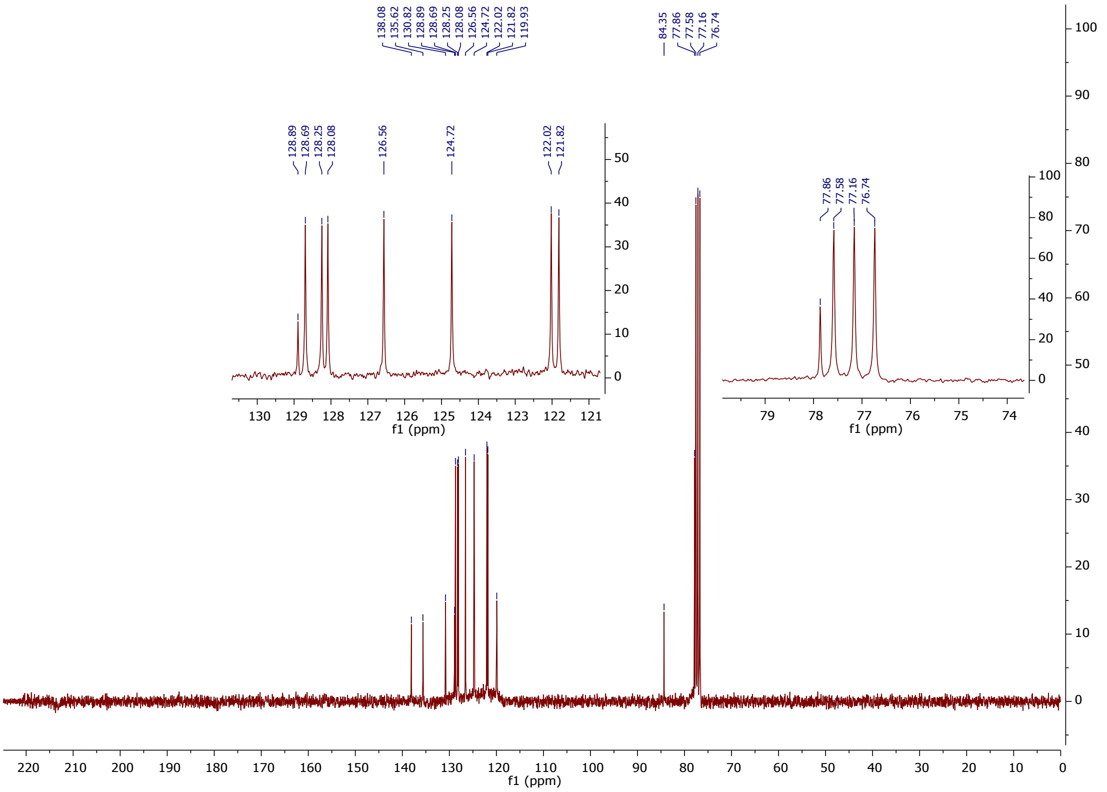

4.7. 8-Ethynylnaphtho[1,2-b]thiophene (300MHz, $CDCl_3$)

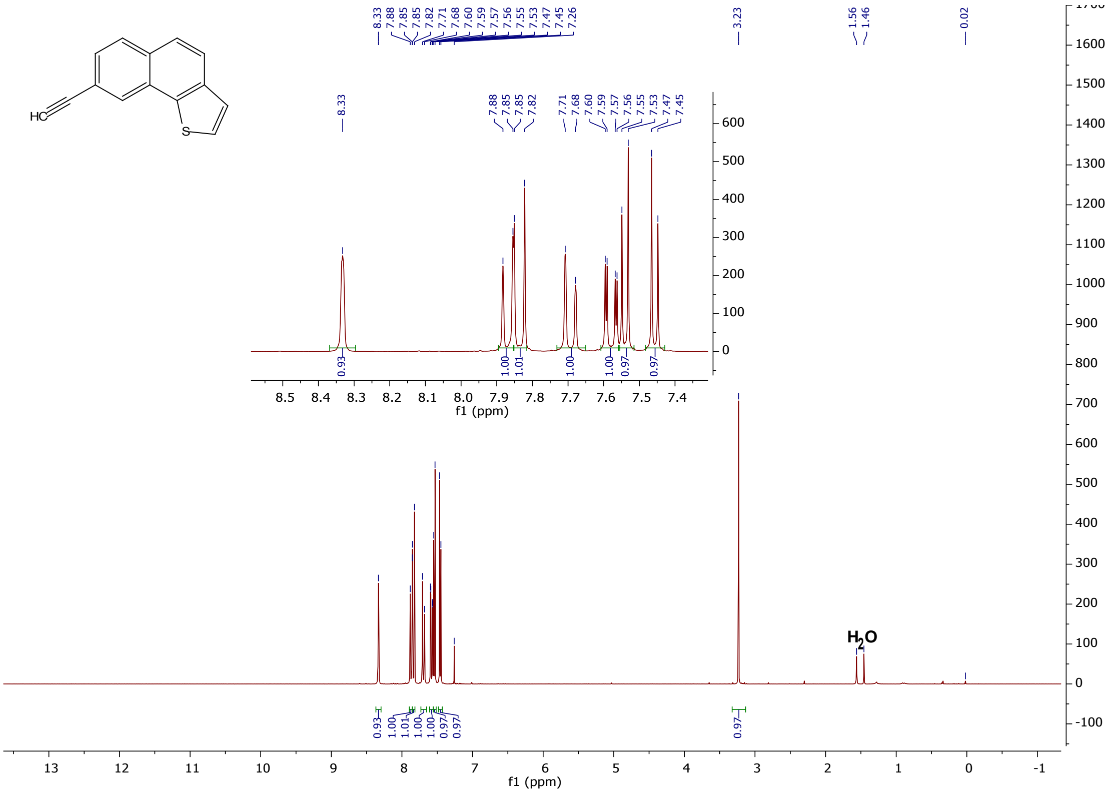

8-Ethynylnaphtho[1,2-b]thiophene (75MHz, $CDCl_3$)

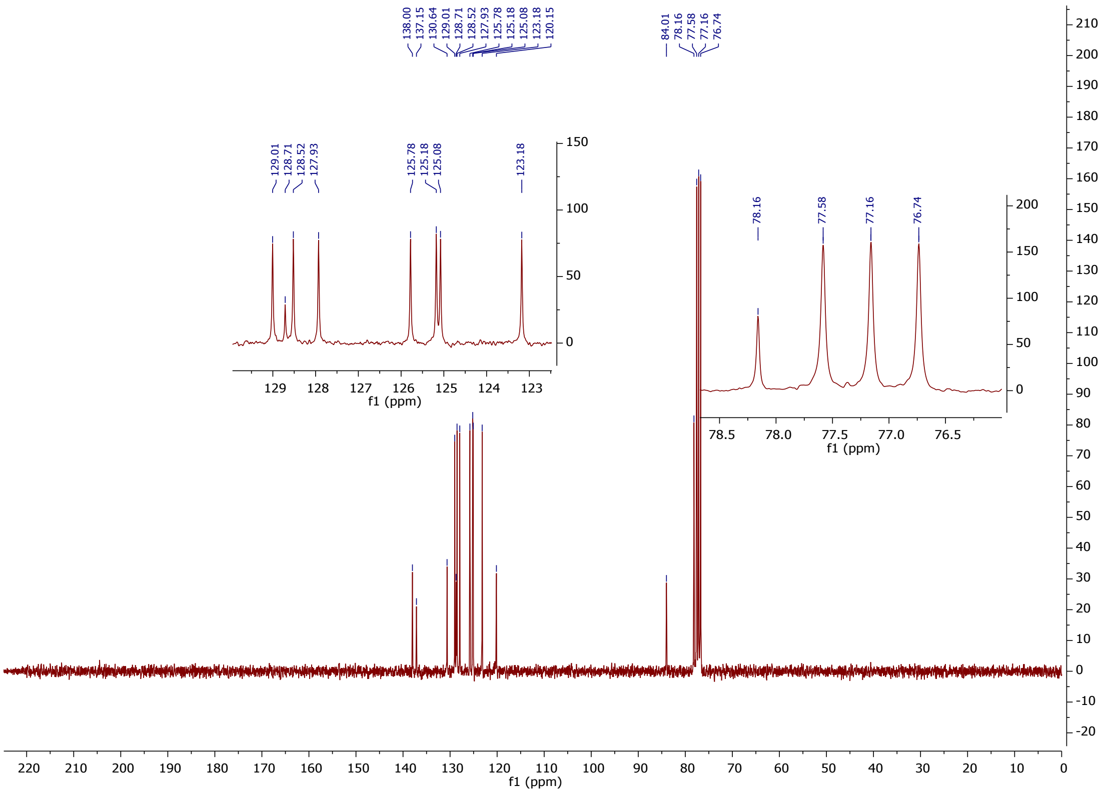

4.8. (*E*)-4,4,5,5-Tetramethyl-2-(2-(naphtho[2,1-b]thiophen-8-yl)vinyl)-1,3,2-dioxoborolane (300MHz, $CDCl_3$)

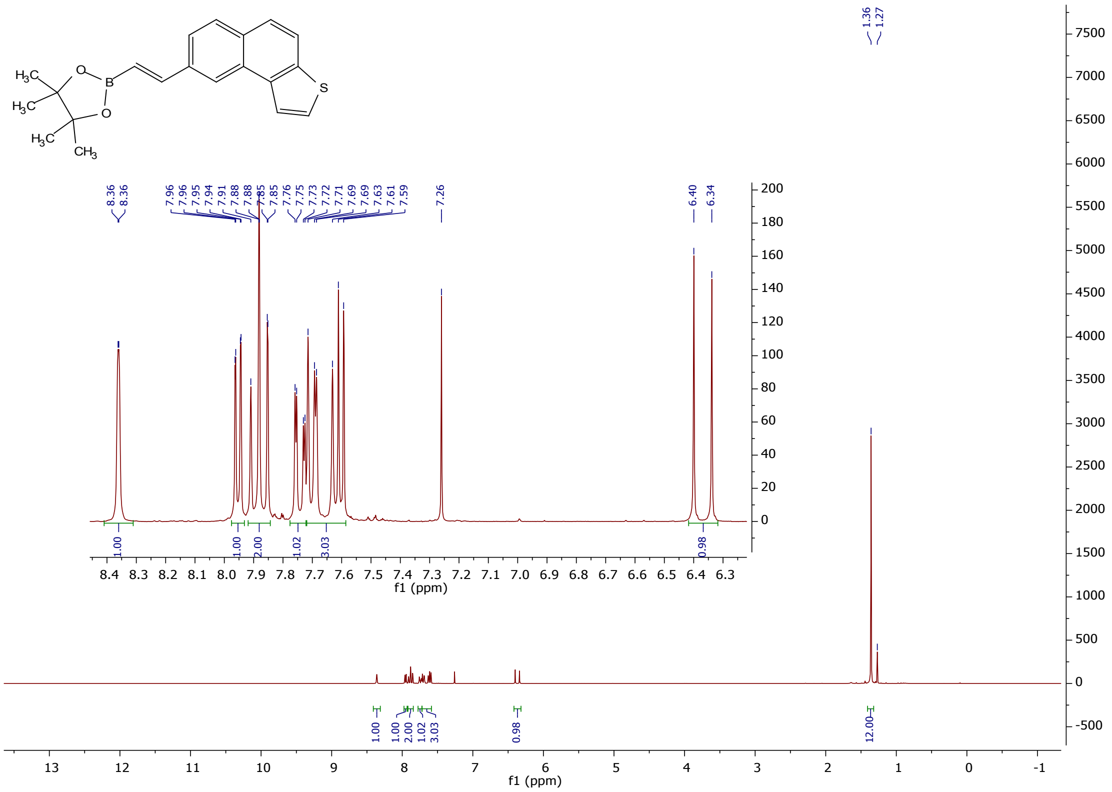

(*E*)-4,4,5,5-Tetramethyl-2-(2-(naphtho[2,1-b]thiophen-8-yl)vinyl)-1,3,2-dioxoborolane (75MHz, $CDCl_3$)

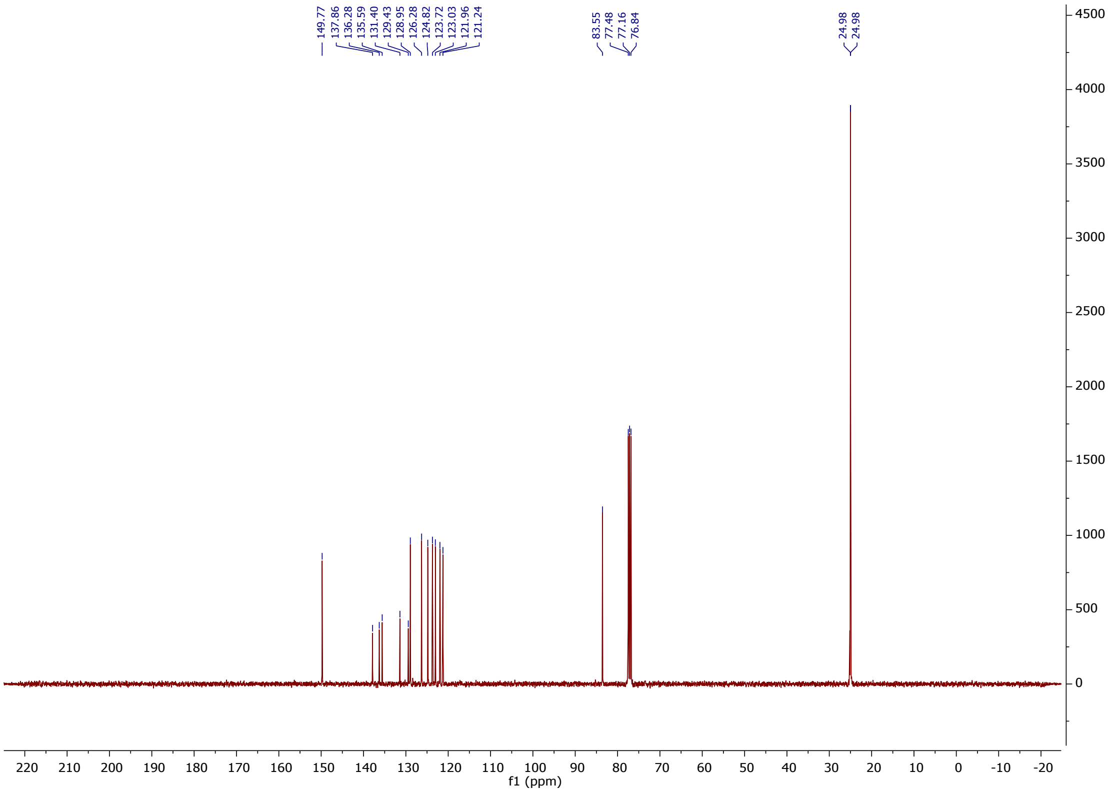

4.9. (*E*)-4,4,5,5-tetramethyl-2-(2-(naphtho[1,2-b]thiophen-8-yl)vinyl)-1,3,2-dioxoborolane (300MHz, $CDCl_3$)

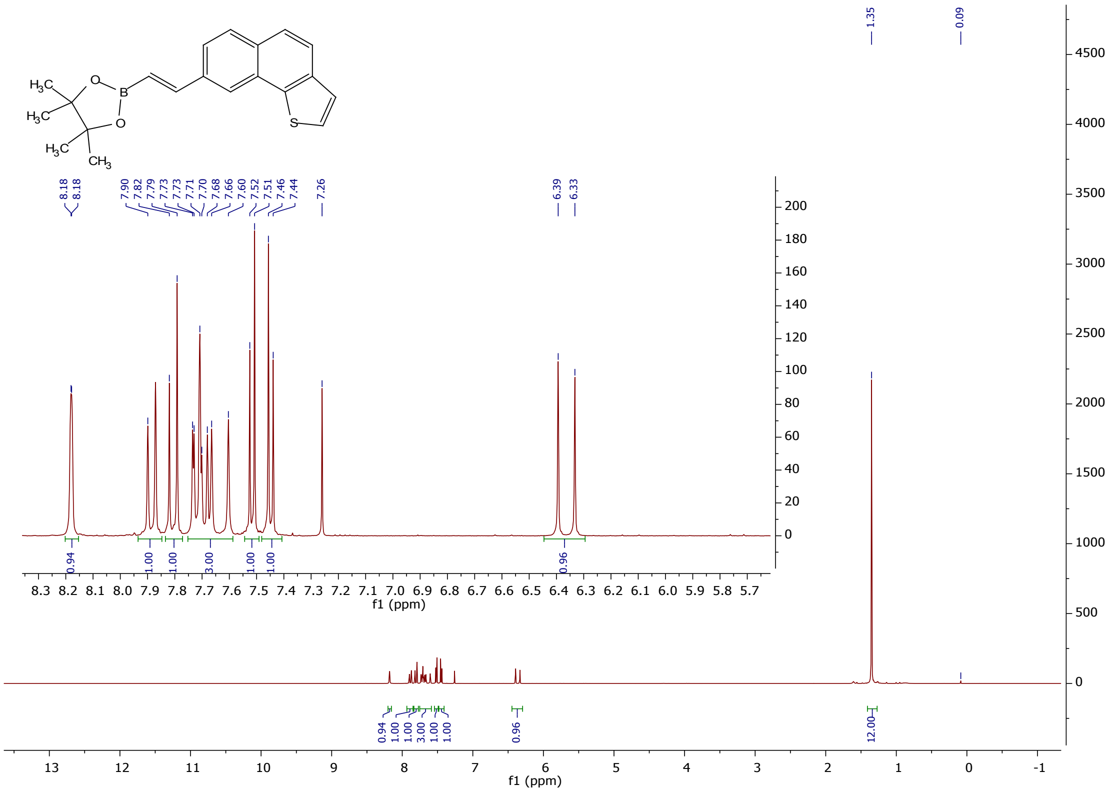

(*E*)-4,4,5,5-Tetramethyl-2-(2-(naphtho[1,2-b]thiophen-8-yl)vinyl)-1,3,2-dioxoborolane (75MHz, $CDCl_3$)

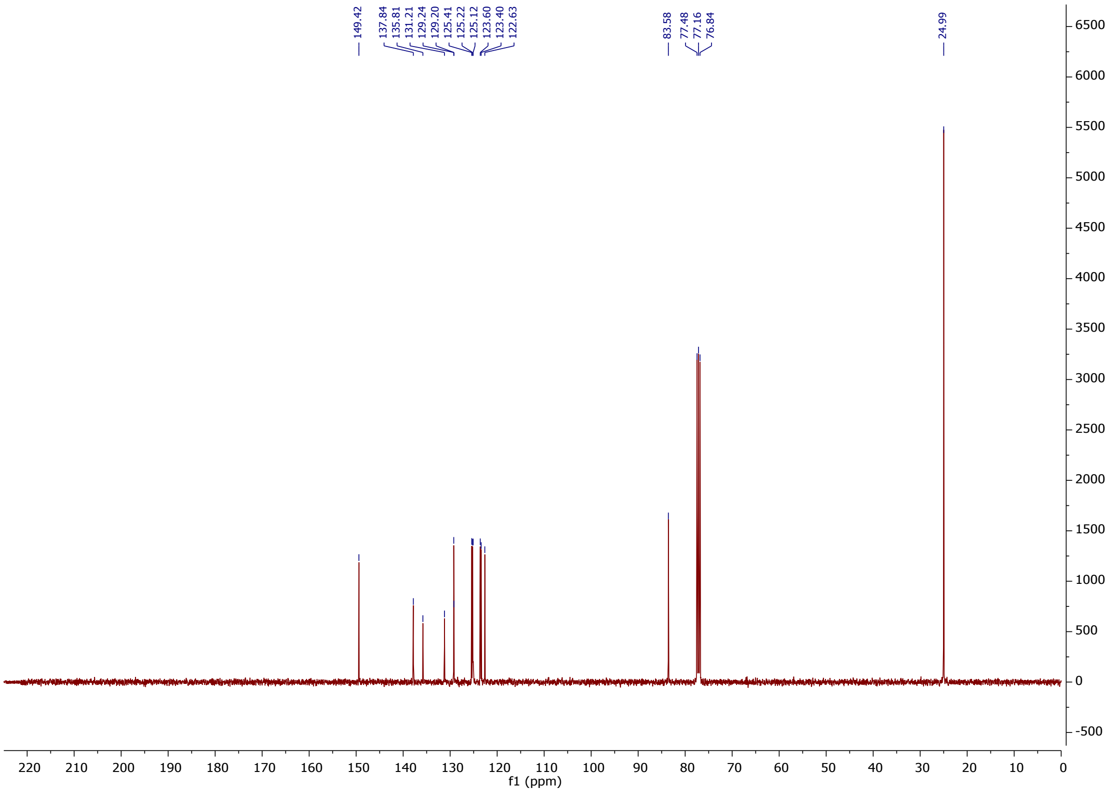

4.10. (*E*)-1,2-Bis(naphto[2,1-b]thiophene-8-yl)ethene (300MHz, $CDCl_3$)

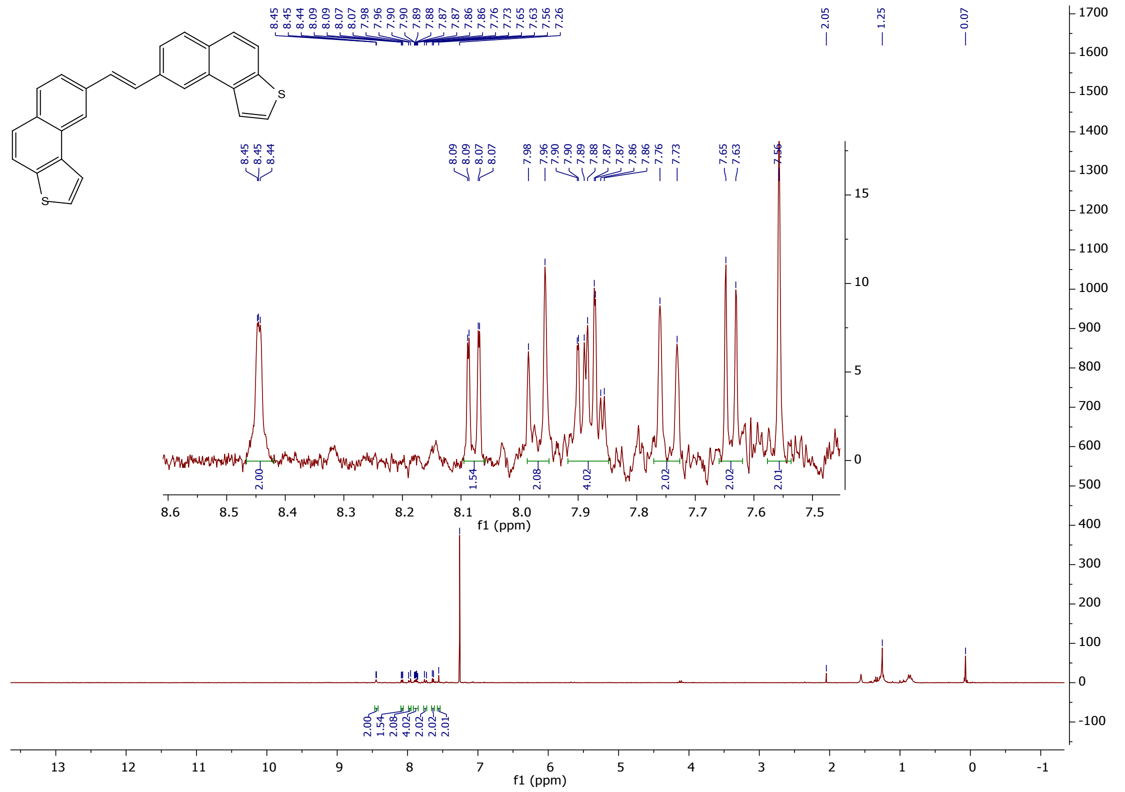

4.11. (*E*)-1,2-Bis(naphto[1,2-b]thiophene-8-yl)ethene (300MHz, $CDCl_3$)

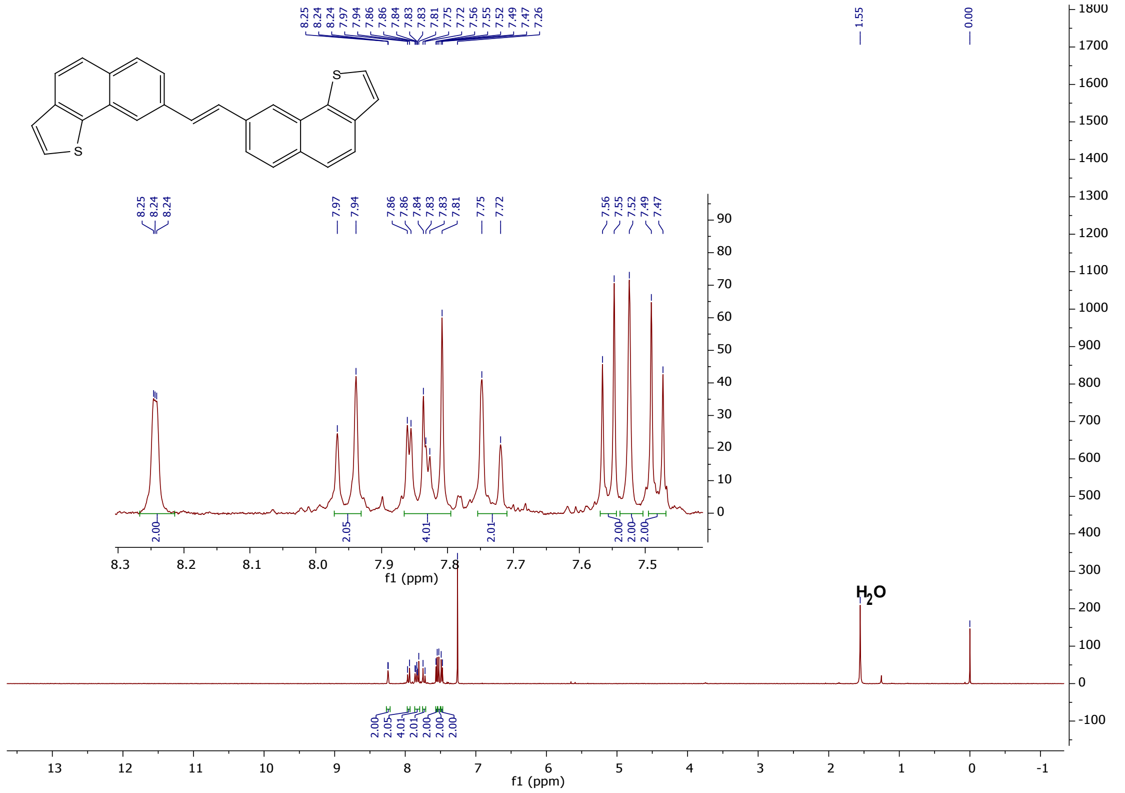

4.12. *Exo*-dithia[7]helicene-**1** (300MHz, $CDCl_3$)

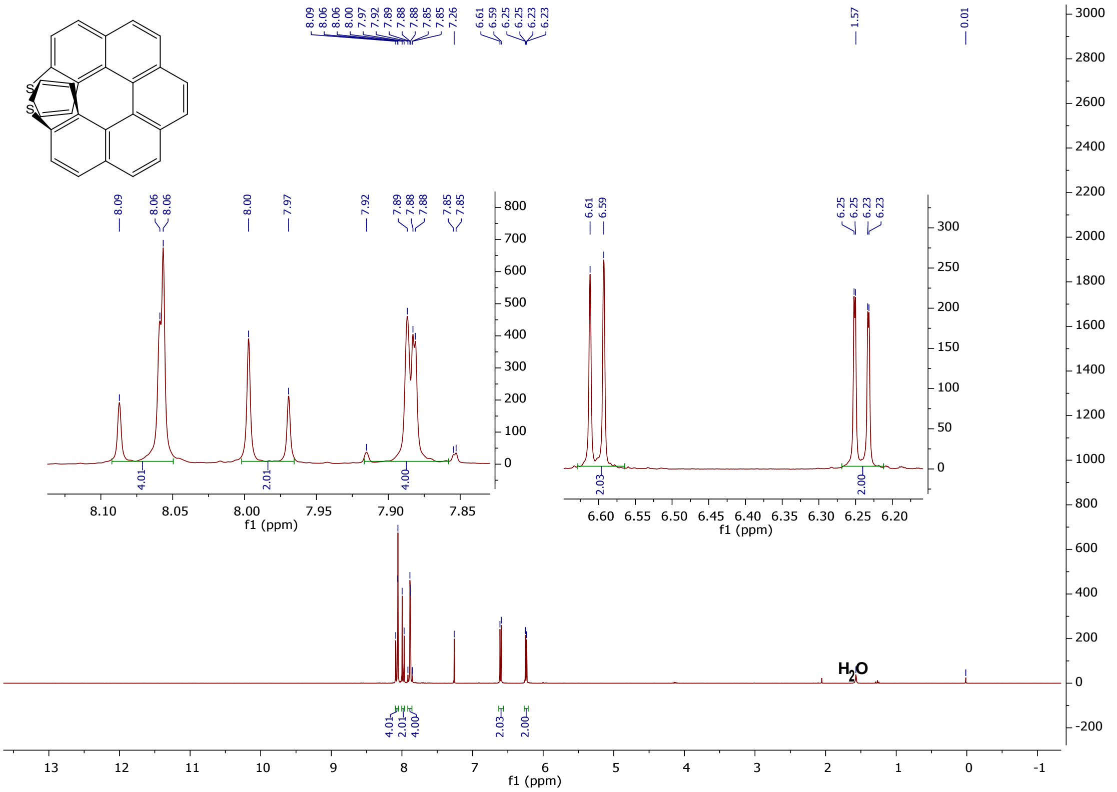

Exo-dithia[7]helicene-**1** (75MHz, $CDCl_3$)

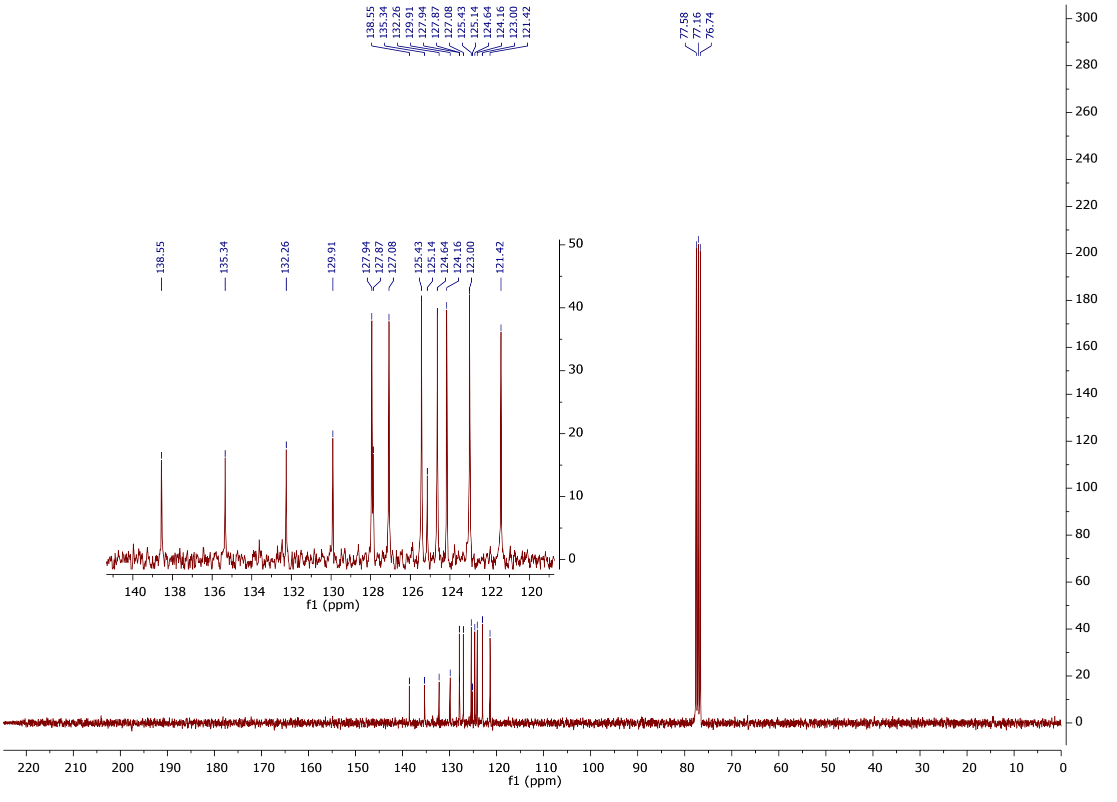

4.13. *Endo*-dithia[7]helicene-**2** (300MHz, $CDCl_3$)

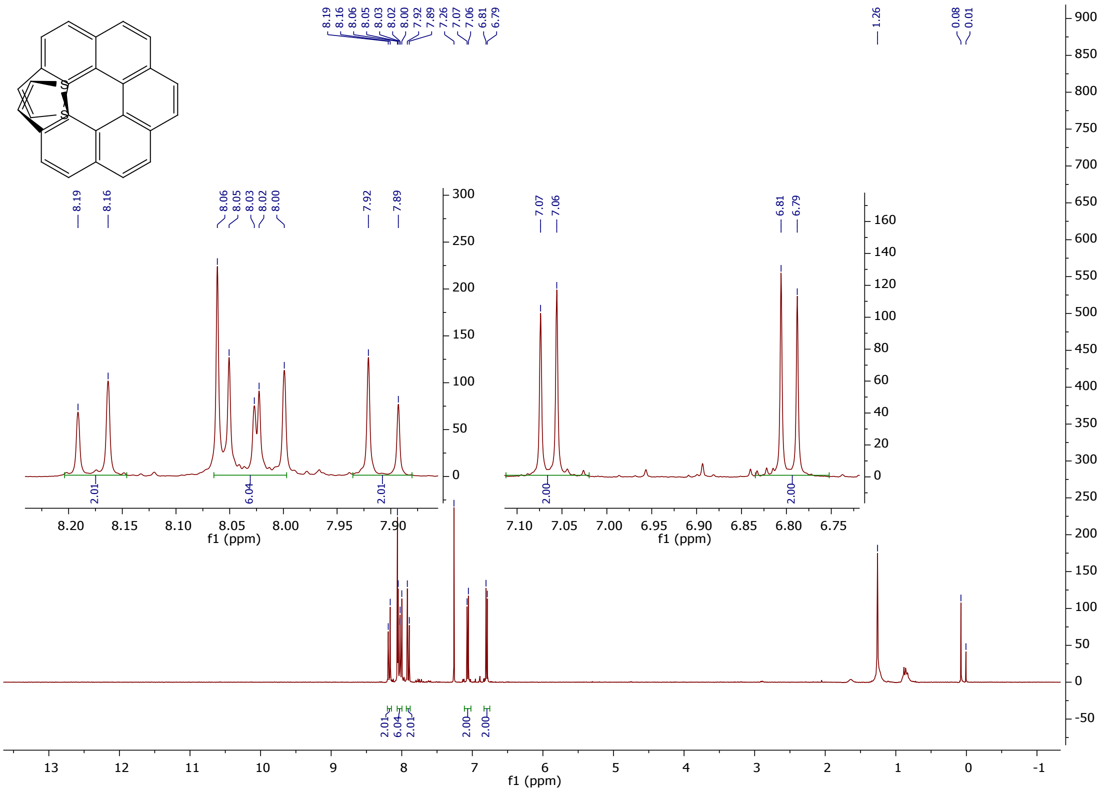

Endo-dithia[7]helicene-2 (75MHz, $CDCl_3$)

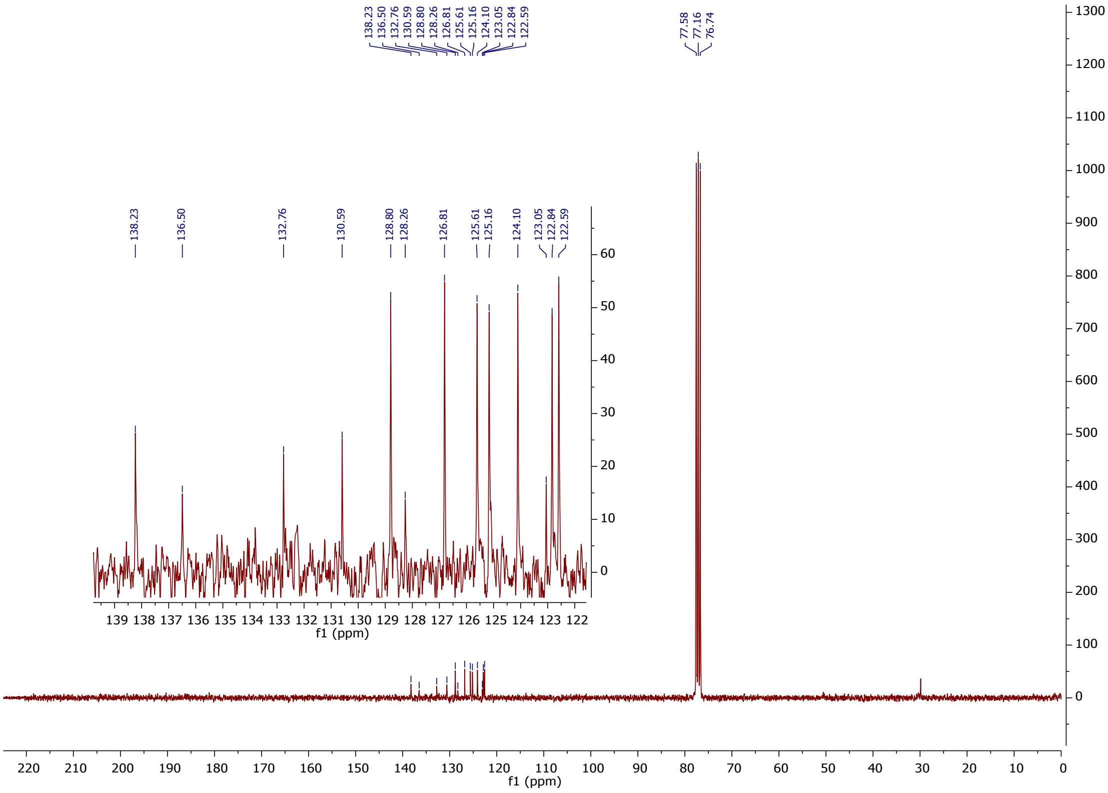

5. X-Ray diffraction (XRD) analysis

5.1. XRD Technical information XX

The crystals of *exo*-dithia[7]helicene-**1** and *endo*-dithia[7]helicene-**2** were analysed using a Bruker CCD-Apex single crystal X-Ray diffraction kit equipped with an X-Ray tube with Mo anode and KRYOFLEX low temperature equipment. We use SMART and SAINT programs to process the data.[8] The SHELXTL structure package of programs was used to perform the characterization of the crystal.[9] This package includes the necessary programs for the determination of the spatial group, absorption corrections, initial determination of the structure by direct methods or Patterson, graphical representation of the results and experimental data table generated from CIF files and simulation.

Exo-Dithia[7]helicene-1

```
Bond precision: C-C = 0.0032 A
Wavelength=0.71073
Cell: a=8.5983(7) b=15.8995(12) c=13.9577(11)
      alpha=90 beta=102.844(2) gamma=90
Temperature: 298 K
```

	Calculated	Reported
Volume	1860.4 (3)	1860.4 (3)
Space group	P 21/c	P 21/c
Hall group	-P 2ybc	
Moiety formula	$C_{26}H_{16}S_2$	$C_{26}H_{16}S_2$
Sum formula	$C_{26}H_{16}S_2$	$C_{26}H_{16}S_2$
Mr	390.49	390.49
Dx, g cm^{-3}	1.394	1.394
Z	4	4
Mu (mm^{-1})	0.295	0.295
F000	808.0	808.0
F000'	809.25	
h,k,lmax	10,18,16	10,18,16
Nref	3299	3293
Tmin, Tmax	0.880, 0.962	0.877, 0.962
Tmin'	0.838	

```
Correction method= # Reported T Limits: Tmin=0.877 Tmax=0.962
AbsCorr = MULTI-SCAN
Data completeness= 0.998 Theta(max)= 25.040
R(reflections)= 0.0384( 2692) wR2(reflections)= 0.1185( 3293)
S = 1.034 Npar= 253
```

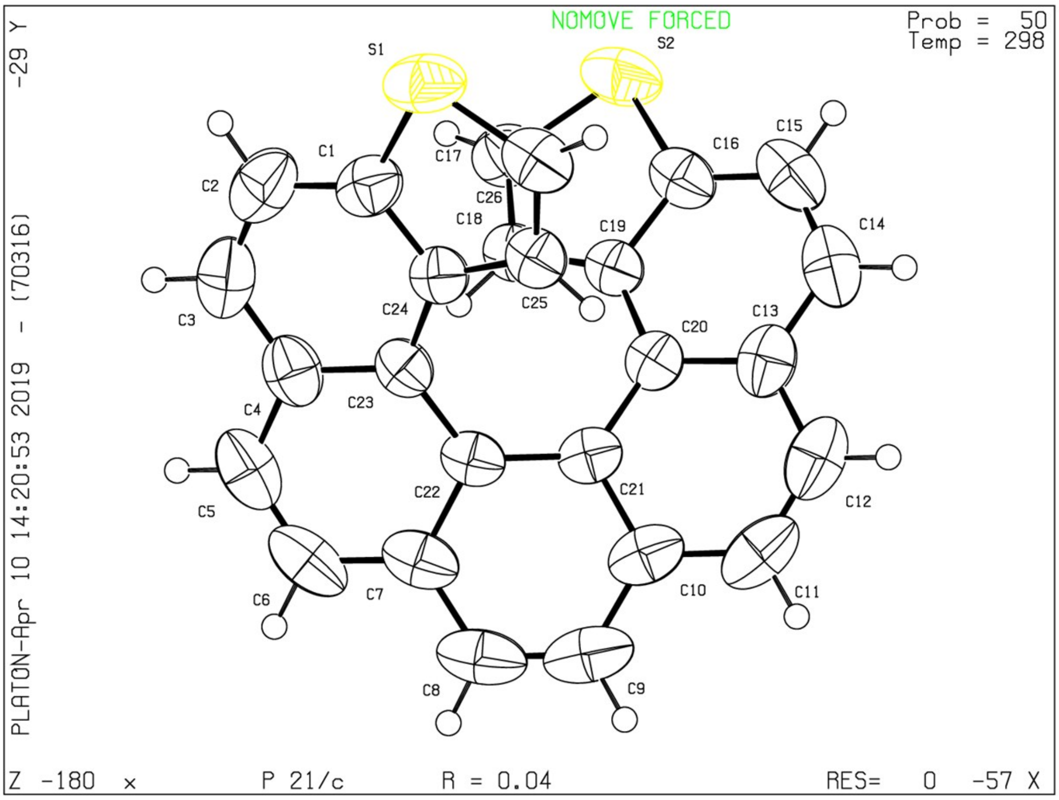

***Endo*-dithia[7]helicene-2**

```
Bond precision: C-C = 0.0036 A
Wavelength=0.71073
Cell: a=13.5378(10) b=13.3994(9) c=20.4288(15)
      alpha=90 beta=90 gamma=90
Temperature: 298 K
```

	Calculated	Reported
Volume	3705.8 (5)	3705.8 (5)
Space group	P b c a	Pbca
Hall group	-P 2ac 2ab	
Moiety formula	$C_{26}H_{16}S_2$	$C_{26}H_{16}S_2$
Sum formula	$C_{26}H_{16}S_2$	$C_{26}H_{16}S_2$
Mr	390.49	390.49
Dx, g cm^{-3}	1.400	1.400
Z	8	8
Mu (mm^{-1})	0.296	0.296
F000	1616.0	1616.0
F000’	1618.51	
h,k,lmax	16,15,24	16,15,24
Nref	3281	3275
Tmin, Tmax	0.918, 0.977	0.865, 0.977
Tmin’	0.847	

```
Correction method= # Reported T Limits: Tmin=0.865 Tmax=0.977
AbsCorr = MULTI-SCAN
Data completeness= 0.998 Theta(max)= 25.060
R(reflections)= 0.0410( 2732) wR2(reflections)= 0.1206( 3275)
S = 1.011 Npar= 253
```

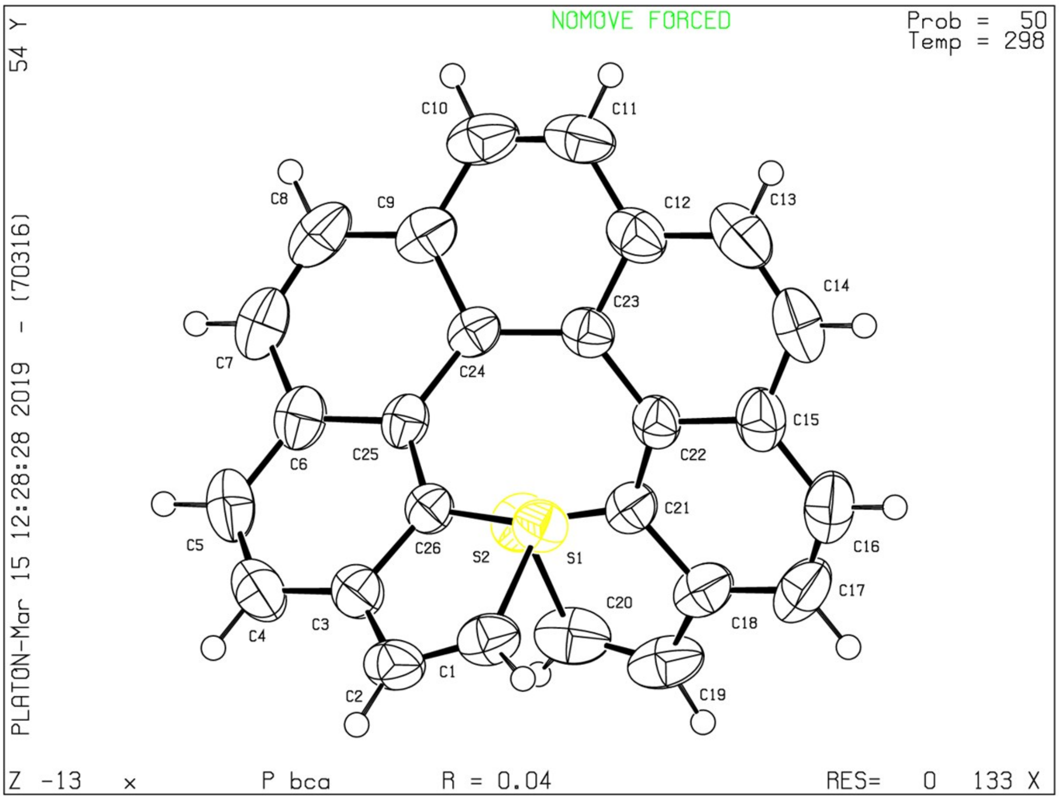

5.2 Geometrical details of the heterochiral molecular pair in the crystal

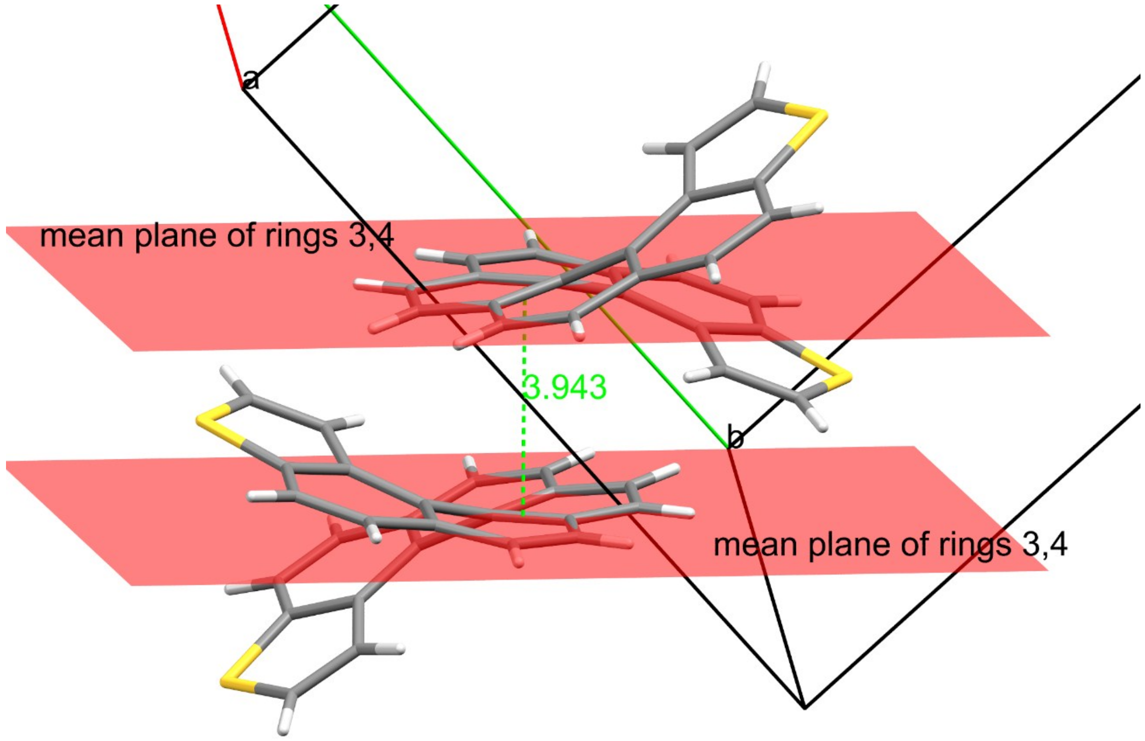

Figure S1. Detail of the π-π interaction in a heterochiral (*P*/*M*) pair of molecules of *exo*-**1**. The interaction occurs by overlap of rings 3-4 between two molecules of opposite chirality, with a separation of 3.943 Å between the mean planes described by these rings.

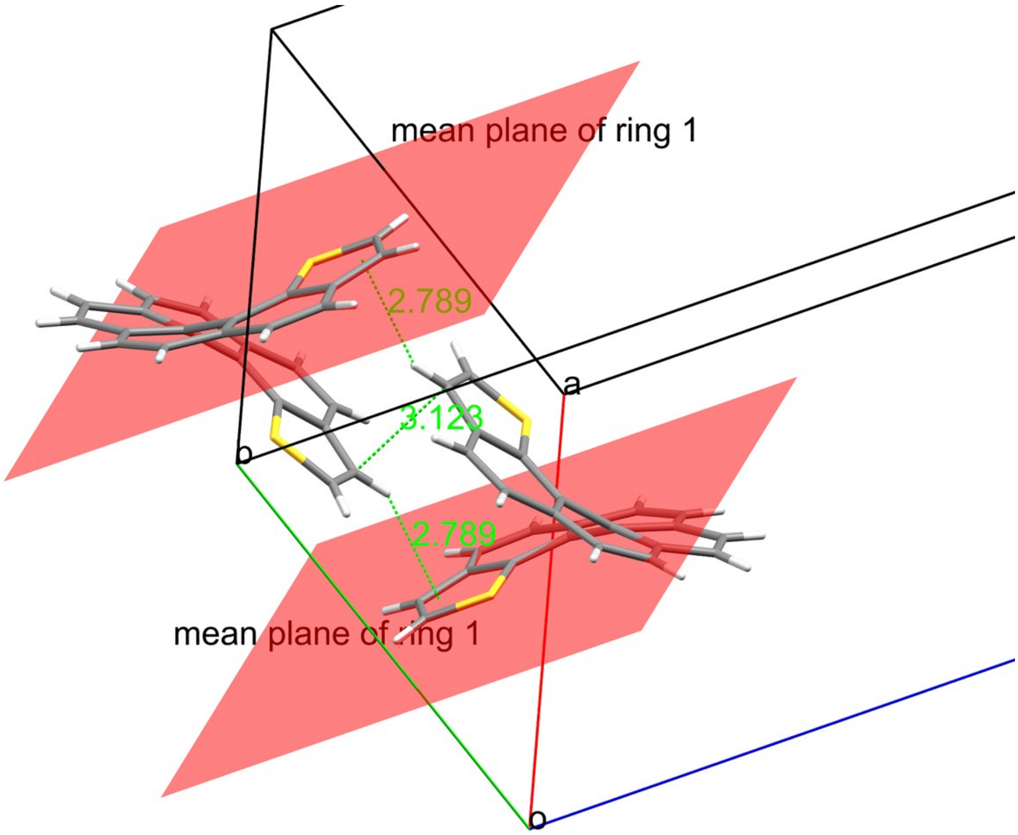

Figure S2. Set of interactions describing a heterochiral pair of molecules in *endo*-**1**. There are two CH-π interactions of 2.789 Å occurring between the CH of carbon 3 and the opposite thiophene π-system. In addition there is a π-π overlap between two carbons 3 in opposite molecules, with a separation of 3.123 Å between them.

6. Differential scanning calorimetry (DSC) and melting point correction applied

DSC is a thermoanalytical technique in which the difference in the amount of heat required to increase the temperature of a sample and reference is measured as a function of temperature. The equipment that we use works between -180ºC and 500ºC and it is equipped with an automatic sample changer with capacity for 50 samples and 5 references. Differential scanning calorimetry was carried out under N_2 atmosphere to check the purity and thermal behavior of *endo*- and *exo*-dithia[7]helicenes.

The melting point for the remaining compounds was measured with a melting point apparatus. To correct the conventional melting point, we made a calibration line using a set of Melting Points Standards from Merck.

mp standard	mp (lit., ºC)	mp (exp., ºC)
1-Heptadecanocarboxylic acid	69-71	63.3-65.2
Benzoic acid	121-123	116.2-116.9
4-Methoxybenzoic acid	182-184	177.4-179.4
Caffeine	235-237	233-235
Anthraquinone	283-286	280-283

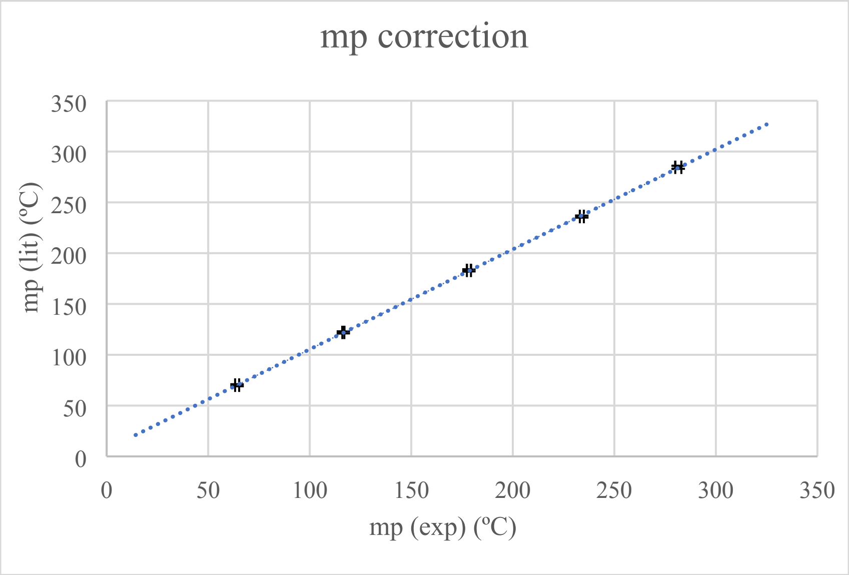

	mp (exp.), (ºC)	mp (corrected), (ºC)
**(*E*)-2-(4-bromostyryl)thiophene**	119,05	124,13
**(*E*)-3-(4-bromostyryl)thiophene**	131,65	136,52
**8-bromonaphtho[2,1-b]thiophene**	104,80	110,11
**8-bromonaphtho[1,2-b]thiophene**	83,50	89,16
**8-ethynylnaphtho[2,1-b]thiophene**	89,15	94,72
**8-ethynylnaphtho[1,2-b]thiophene**	47,90	54,15
**(*E*)-4,4,5,5-tetramethyl-2-(2-(naphtho[1,2-b]thiophen-8-yl)vinyl)-1,3,2-dioxoborolane**	132,70	137,55
**(*E*)-1,2-bis(naphto[2,1-b]thiophene-8-yl)ethene**	310,00	311,95
**(*E*)-1,2-bis(naphto[1,2-b]thiophene-8-yl)ethene**	264,50	267,19

For *exo*-dithia[7]helicene-**1**, DSC shows a sharp endothermic event corresponding to the melting. A mp=231.13ºC is given by the extrapolated onset temperature of the melting peak.

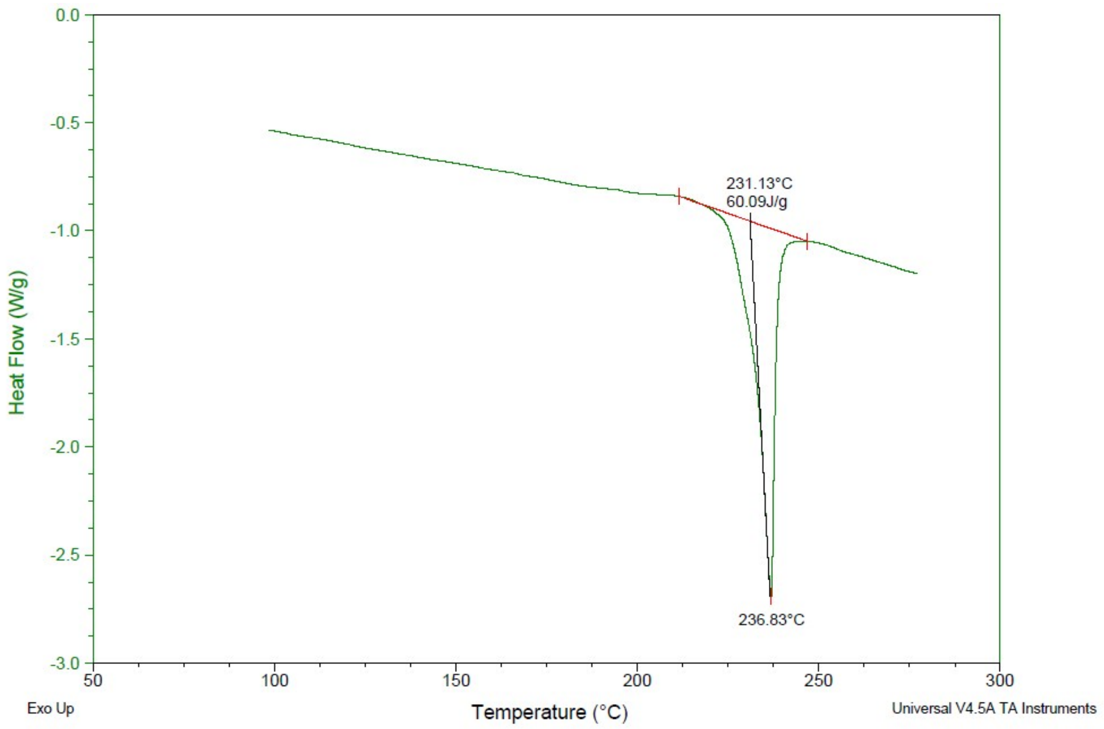

For *endo*-dithia[7]helicene-**2** DSC shows a sharp endothermic event corresponding to the melting, but in this case a slight product decomposition is observed when we reexamined the sample by NMR. A mp=350.29ºC is given by the extrapolated onset temperature of the melting peak.

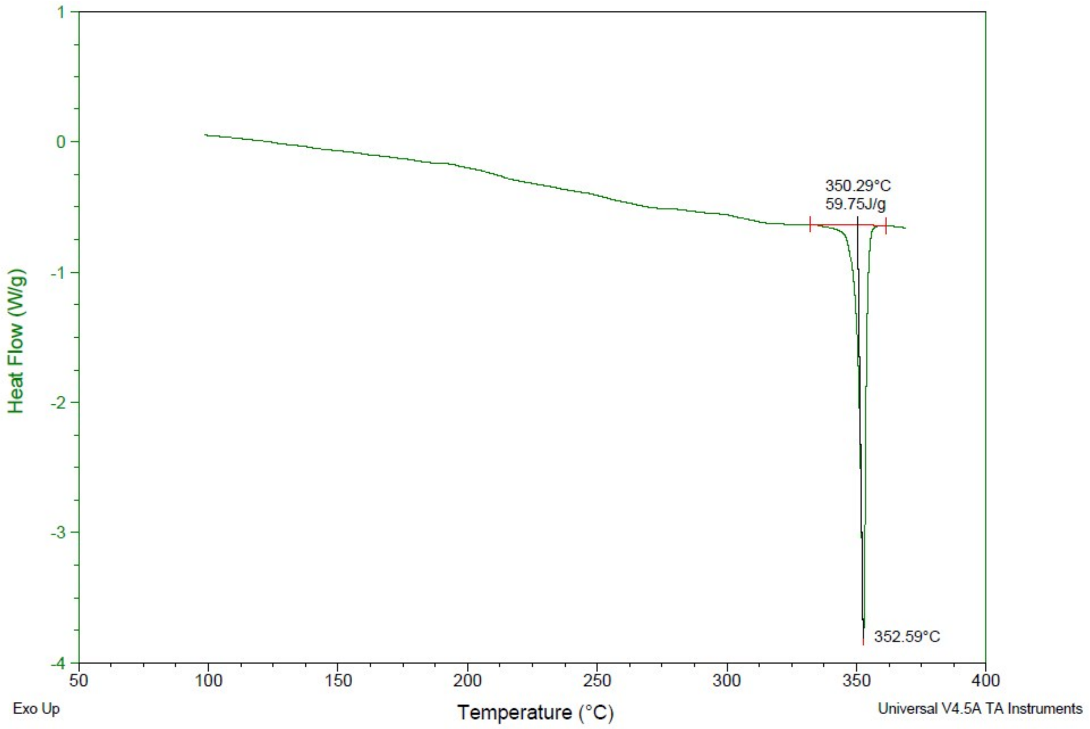

7. HPLC analysis

Chromatographic conditions: **Column**: 00G-4601-E0 (Kinetex 5µm C18 100Å, 250×4.6 mm). **Mobile phase**: Isocratic gradient of acetonitrile/water (80:20) at 25ºC. **Flow rate**: 1 ml/min. **Detection**: UV at 254 nm.

***Exo*-dithia[7]helicene-1**

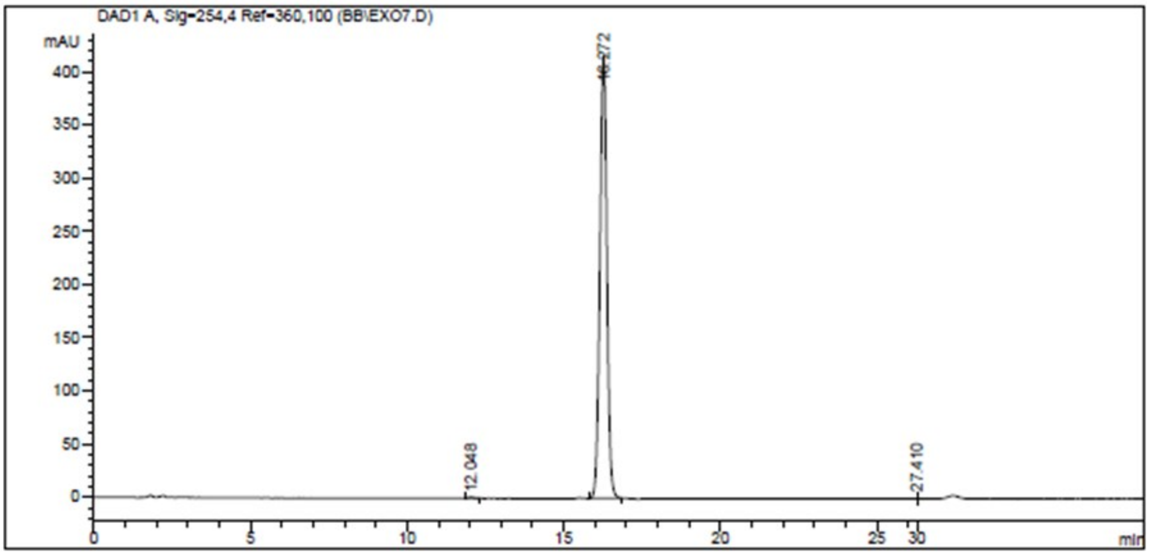


```
================================================================
                    Area Percent Report
================================================================

Sorted By             :      Signal
Multiplier            :      1.0000
Dilution              :      1.0000
Sample Amount         :      1.00000  [ng/ul]   (not used in calc.)
Use Multiplier & Dilution Factor with ISTDs

Signal 1: DAD1 A, Sig-254,4 Ref-360,100
```

Peak #	RetTime [min]	Type	Width [min]	Area [mAU*s]	Height [mAU]	Area %
1	12.048	BB	0.1478	13.68202	1.17557	0.2080
2	16.272	BB	0.2419	6495.96045	416.73294	98.7708
3	27.410	BP	0.2972	67.16333	2.76718	1.0212

***Endo*-dithia[7]helicene-2**

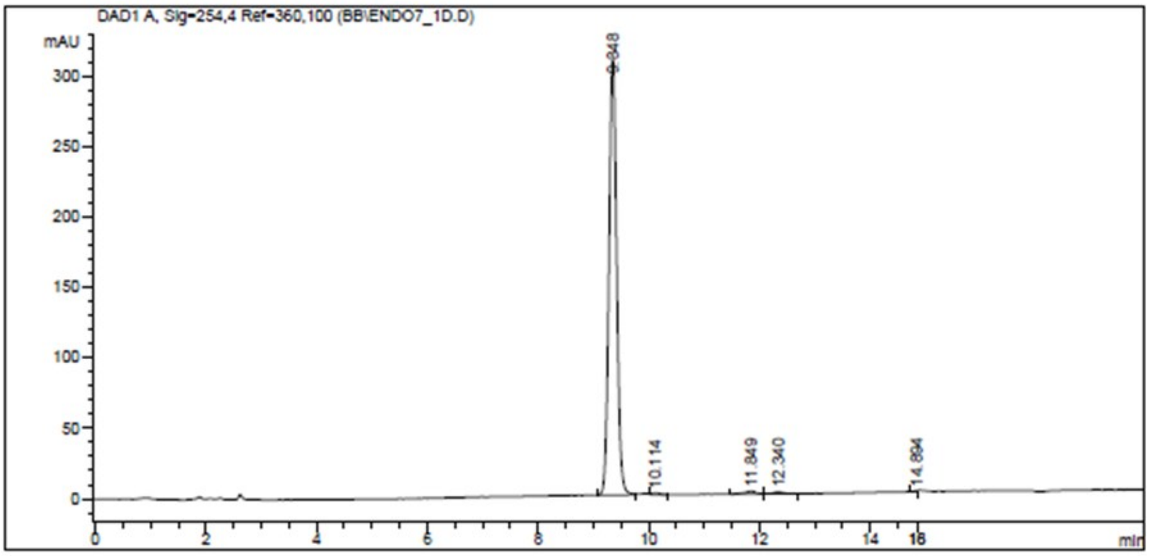


```
=====================================================================
                         Area Percent Report
=====================================================================

Sorted By              :      Signal
Multiplier             :      1.0000
Dilution               :      1.0000
Sample Amount          :      1.00000  [ng/ul]   (not used in calc.)
Use Multiplier & Dilution Factor with ISTDs

Signal 1: DAD1 A, Sig=254,4 Ref=360,100
```

Peak #	RetTime [min]	Type	Width [min]	Area [mAU*s]	Height [mAU]	Area %
1	9.348	BB	0.1480	2996.09985	312.06818	97.7069
2	10.114	BB	0.1332	13.15045	1.30890	0.4289
3	11.849	PV	0.2175	29.77080	1.93492	0.9709
4	12.340	VB	0.1494	14.89701	1.32315	0.4858
5	14.894	BB	0.1705	12.49756	1.00929	0.4076

8. Mass Spectroscopy (MS)

MS for **1** and **2** were carried out using the direct insertion probe (DIP) technique. The list of the molecular ions (*M*+) and main fragmentation peaks (>10% abundance) can be found in Section 3, Characterization of compounds.

***Exo*-dithia[7]helicene-1**

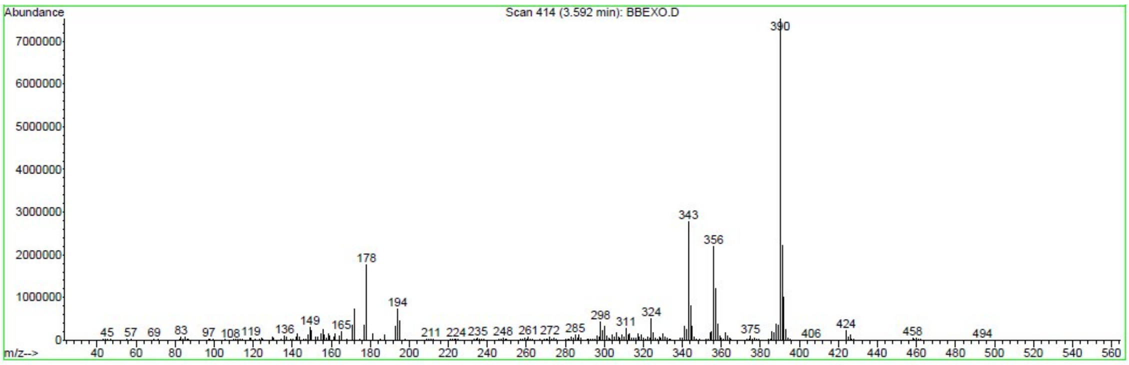

***Endo*-dithia[7]helicene-2**

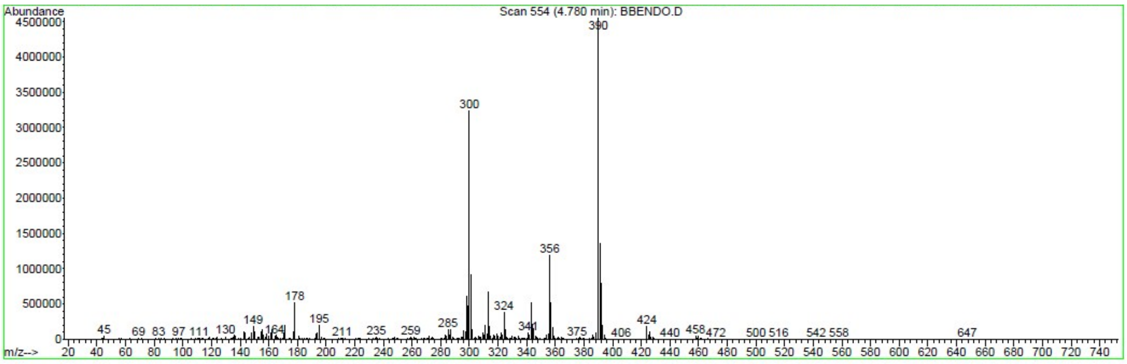

9. UV-vis Spectroscopy

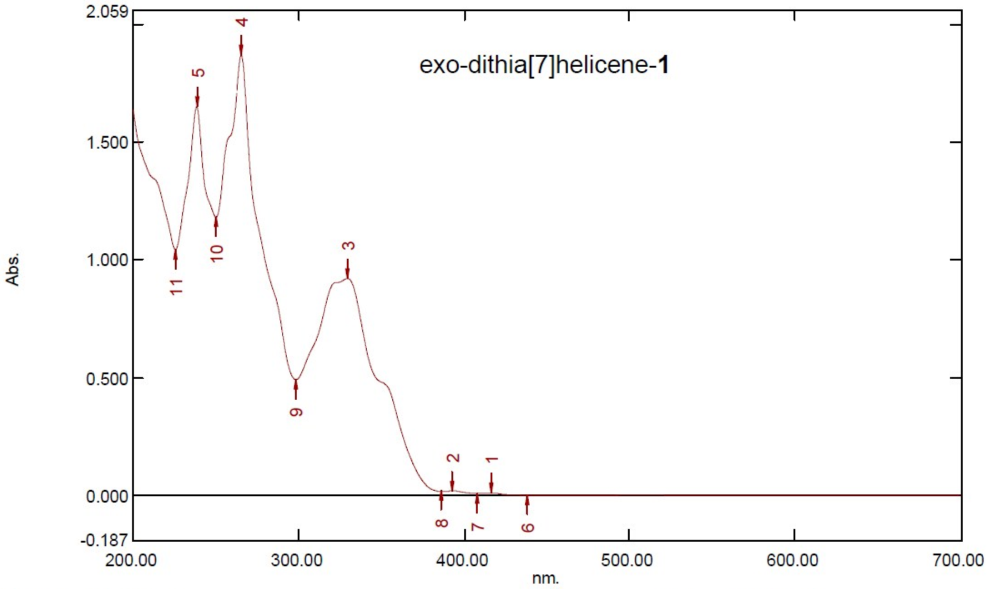

Measurement Properties	
Wavelength Range (nm.):	200,00 to 700,00
Scan Speed:	Medium
Sampling Interval:	0,1
Auto Sampling Interval:	Disabled
Scan Mode:	Single
Sample Preparation Properties	
Concentration:	$3{\cdot}10^{-5}$ M
Solvent:	n-hexane
Path Length:	10 mm
Additional Information:	

No.	P/V	Wavelength	Abs.	Description
1	↑	416.70	0.009	
2	↑	392.90	0.019	
3	↑	329.40	0.922	
4	↑	265.30	1.872	
5	↑	238.50	1.653	
6	↓	438.40	0.001	
7	↓	407.60	0.008	
8	↓	386.60	0.016	
9	↓	298.40	0.492	
10	↓	250.00	1.182	
11	↓	225.60	1.044	

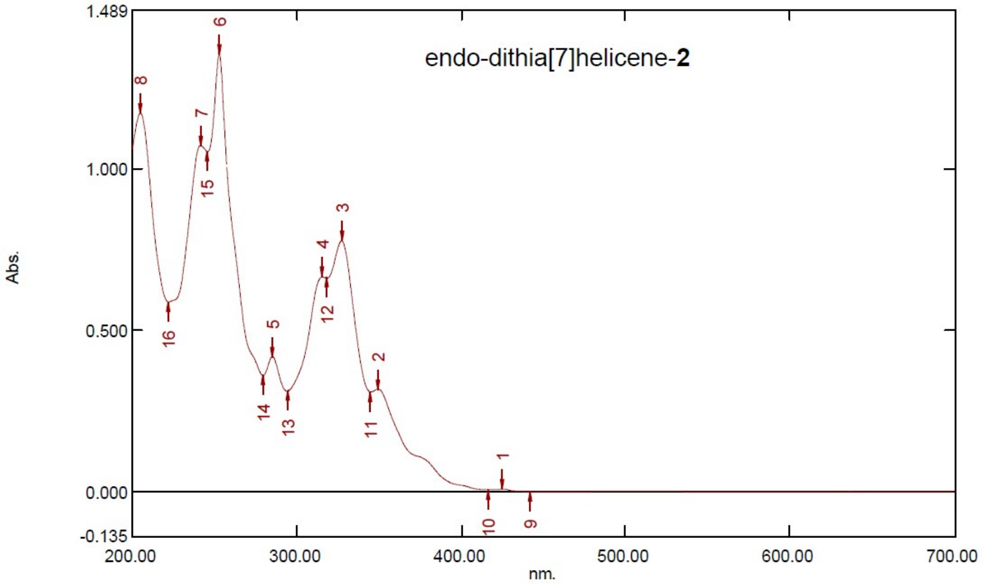

Measurement Properties
Wavelength Range (nm.): 200,00 to 700,00
Scan Speed: Medium
Sampling Interval: 0,1
Auto Sampling Interval: Disabled
Scan Mode: Single

Sample Preparation Properties

Concentration: $2.5 \cdot 10^{-5}$ M
Solvent: n-hexane

Path Length: 10 mm
Additional Information:

No.	P/V	Wavelength	Abs.	Description
1	↑	425.30	0.008	
2	↑	349.20	0.317	
3	↑	327.30	0.777	
4	↑	315.50	0.665	
5	↑	285.20	0.418	
6	↑	252.80	1.353	
7	↑	241.30	1.071	
8	↑	204.80	1.172	
9	↓	442.30	0.000	
10	↓	416.00	0.006	
11	↓	345.00	0.309	
12	↓	318.30	0.660	
13	↓	294.10	0.311	
14	↓	279.40	0.359	
15	↓	245.80	1.052	
16	↓	222.00	0.587	

10. Scanning Tunneling Microscopy (STM)

10.1. Procedure to deposit molecules over the substrate

Exo- and *endo*-dithia[7]helicene (*exo*-**1** and *endo*-**2**) molecules were studied on flame-annealed gold (111) deposited on glass. A homebuilt STM was employed controlled with a Dulcinea Control Unit from Nanotec and the WSxM 5.0 Develop 7.0.[10]

The molecules were deposited over flame annealed gold(111) substrates (by Arrandee tm[11]). The substrates were prepared right before the deposition of the molecules as follows: First the substrates were immersed in piranha solution (3:1 of sulfuric acid and hydrogen peroxide). The bath duration was around $t \approx 300$ s, followed by an immersion with ultrapure miliQ water to remove the organic residues off the surface. The substrates were dried under argon gas. Finally, to prepare the atomically flat (111) surfaces, the substrate was thermally annealed using a flame torch with a mixture butane/propane during $t \approx 210$ s.

10.2. SMT images of *exo*-dithia[7]helicene-**1** and *endo*-dithia[7]helicene-**2** with another concentration

Exo-**1** and *endo*-**2** 0.2 mg ml^{-1}

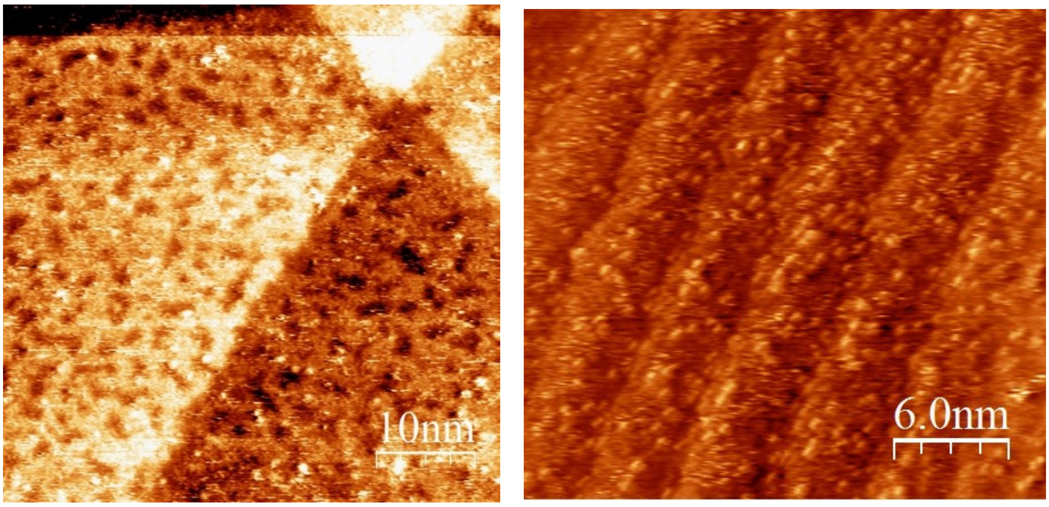

Figure S3. Topographic images of *exo*-dithia[7]helicene and *endo*-dithia[7]helicene molecules at room temperature in a concentration of 0.2 $mg\,ml^{-1}$ deposited by drop casting method. (a) Topographic image of exo-DTE molecules with a set point I_t = 1.61 nA and V_{bias} = −0.478 V. (b) Topographic image of isolated endo-DTE molecules with parameters I_t = 0.18 nA and V_{bias} = −0.591 V.

10.3. Illustration of the geometries of *exo*-dithia[7]helicene-**1** and *endo*-dithia[7]helicene-**2**

Figure S4 shows values of some of the dimensions of the molecules *exo*-dithia[7]helicene-**1** and *endo*-dithia[7]helicene-**2**.

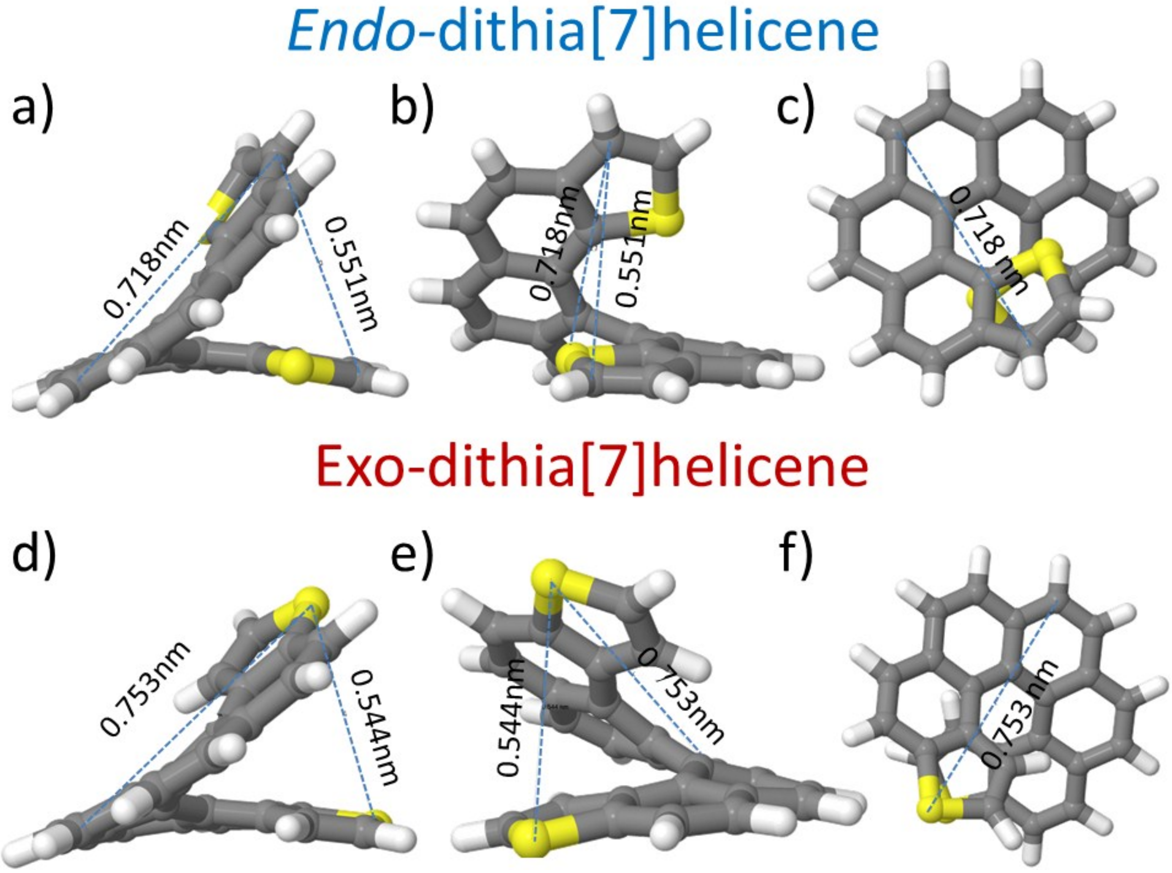

Figure S4. Panels a),b) and c) illustrate in different angles of observation the *endo*-dithia [7]helicene-**1**. Panels d), e) and f) illustrate *exo*-dithia[7]helicene-**2**, different angles of observation also. Dashed lines illustrate the distance between different atoms.

11. References

---

[1] M. Urbani, K. Ohkubo, D. M. Shafiqul Islam, S. Fukuzumi and F. Langa, *Chem. Eur. J.,* 2012, **18**, 7473-7485.
[2] S. Moussa, F. Aloui and B. Ben Hassine, *Synth. Comm.* 2013, **43**, 268-276.
[3] C.-C. Chen, K. Pan, S.-L. Wang and T.-I. Ho, *J. Lumin.* 1997, **71**, 321-328.
[4] P. A. Wender, A. B. Lesser and L. E. Sirois, *Angew. Chem*. 2012, **124**, 2790-2794.
[5] L. Ji-Eon, J. Kwon and J. Yun, J. *Chem. Commun.*, 2008, 733-734.
[6] G. A. Molander and A. R. Brawn, *J. Org. Chem*., 2006, **71**, 9681-9686.
[7] S. Moussa, F. Aloui, B. Ben Hassine, *Synth. Comm.* 2011, **41**, 1006-1016.
[8] *SAINT* version 6.02A: *Area-Detector Integration Software*; Siemens Industrial Automation Inc.: Madison, WI, 1995.
[9] *SHELX97* [Includes *SHELXS97*, *SHELXL97* and *CIFTAB*]—*Programs for Crystal Structure Analysis* (Release 97-2). Sheldrick, G. M., Institüt für Anorganische Chemie der Universität: Tammanstrasse 4, D-3400 Göttingen, Germany, 1998.
[10] I. Horcas et al. Rev. Sci. Instrum. 2007, **78**, 013705.
[11] Arrandee tm. Gold Arrandee – Au (111). https://www.arrandee.com/.
